# Antigen-experienced airway CD8^+^ effector memory T cells promote a detour pathway for mycobacterial killing in the spleen

**DOI:** 10.1126/sciadv.aec7792

**Published:** 2026-08-21

**Authors:** Julia Seifert, Visai Muruganandah, Socorro Miranda-Hernandez, Harindra D. Sathkumara, Ana Maria Valencia-Hernandez, Saparna Pai, Andreas Kupz

**Affiliations:** ^1^ Australian Institute of Tropical Health and Medicine, James Cook University, Cairns and Townsville, Queensland, Australia.; ^2^ TAARA Therapeutics Pty Ltd, Cairns, Queensland, Australia.

## Abstract

A 3-week delay in the onset of T cell immunity to *Mycobacterium tuberculosis* (*Mtb*) infection compromises lung bacterial control, resulting in tuberculosis (TB). Restoring T cell function is a priority. We identified how airway CD44^+^CD62L^−^KLRG1^+^CD8^+^ effector memory T cells (T_EM_) in the lung enhance mycobacterial clearance via the spleen. Unexpectedly, a secondary immune response following repeated exposure of mice to Bacille Calmette-Guérin generated lower numbers of T_EM_ versus primary. Paradoxically, lower numbers correlated with faster bacterial clearance, due to enhanced trafficking of secondary T_EM_ to the spleen, where they responded to antigen. Further, secondary T_EM_ recruited dendritic cells, which accelerated bacterial translocation from the lung to the spleen. Treating mice with a threshold number of airway-derived secondary T_EM_ significantly reduced *Mtb* burden in the lung and spleen. Notably, *Mtb* killing was expedited by at least 15 days. Repeated antigen encounter bypassed priming requirements and enhanced T_EM_ durability, uncovering a spleen-centered protective mechanism with implications for improved TB vaccines and therapies.

## INTRODUCTION

Tuberculosis (TB) is the leading cause of death worldwide from a single infectious organism with more than 10 million new cases and 1.3 million deaths in 2023 (*1*). Despite widespread use, Bacille Calmette-Guérin (BCG), the only licensed vaccine against TB, provides inconsistent protection against pulmonary TB (*2*), and multiple TB vaccine candidates have failed to surpass BCG efficacy in clinical trials (*3*, *4*). A major barrier to developing superior vaccines and therapeutics is the incomplete mechanistic understanding of BCG vaccination and *Mycobacterium tuberculosis* (*Mtb*) infection and the resultant immunological protection against TB, particularly during the early stages of infection.

CD8^+^ T cells are necessary for control of chronic *Mtb* infection (*5*), and memory subsets, including central, effector, and resident memory cells (T_CM_, T_EM_, and T_RM_ respectively; Table 1) protect against murine TB (*6*, *7*). Phenotypic markers such as CD127 and CD62L reliably distinguish CD8^+^ T_CM_, T_EM_, and T_RM_ (*8*, *9*); however, their precise phenotype and function in protection against TB remain unclear (*10*). Because T_EM_ as potent effectors produce high levels of interferon-γ (IFN-γ), they have been a key target of early TB vaccine development, yet evidence suggests they are ineffective in TB control. Accordingly, vaccine-induced IFN-γ levels do not correlate with protection. T_EM_ are short lived under persistent antigen exposure and, therefore, inefficiently recruited to the lung early after infection (*11*). During chronic TB, they accumulate in the lung vasculature rather than the parenchyma, limiting robust site-specific response (*11*). However, this does not mean that long-lived cells are better at inducing immediate protective immunity—the intended goal of vaccination, as T_CM_ are long lived but show limited effector function. Thus, neither T_EM_ nor T_CM_ fulfil both criteria of memory: longevity and rapid effector function (*12*). This may explain why several vaccination strategies induce poor protection despite the generation of antigen-specific memory T cells.

**Table 1.** Phenotypic characteristics of mouse memory CD8^+^ T cells.

Cell type										
Memory T cells	CD45^+^	CD3^+^	CD44^hi^	CD62L^lo^						
Effector T cells	CD45^+^	CD3^+^	CD44^lo^	CD62L^lo^		KLRG1^lo^				
T effector memory cells (T_EM_)	CD45^+^	CD3^+^	CD44^hi^	CD62L^lo^	CD103^−^	KLRG1^+^				
T central memory cells (T_CM_)	CD45^+^	CD3^+^	CD44^hi^	CD62L^hi^						
T resident memory cells (T_RM_)	CD45^+^	CD3^+^	CD44^hi^	CD62L^lo^	CD103^+^	KLRG1^−^				
Short-lived effector cells (SLEC)	CD45^+^	CD3^+^	CD44^hi^	CD62L^lo^	CD103^−^	KLRG1^+^	CD27^lo^	CD127^lo^	CD43^+^	T-bet^+^
Memory precursor effector cells (MPEC)	CD45^+^	CD3^+^	CD44^hi^	CD62L^lo^	CD103^−^	KLRG1^lo^	CD27^+^	CD127^hi^	CD43^lo^	T-bet^lo^

In contrast to other pathogens, the onset of T cell immunity to *Mtb* infection and BCG vaccination in the lung is delayed by about 2 to 3 weeks (*13*). While different reasons for this delay have been proposed, including interference with major histocompatibility complex (MHC)-mediated antigen presentation and delayed trafficking of antigen-presenting cells (APCs) (*14*), the exact sequence of events leading to the delay remains unclear. Nevertheless, there is consensus that *Mtb* subverts T cell immunity in the lung, promoting its own replication and dissemination, as well as disease progression (*15*). Strategies that enhance CD8^+^ T cell responses in the lung could undoubtedly help develop better vaccine and therapeutic strategies. Key unresolved questions include (i) when and how protective CD8^+^ T cell immunity to mycobacteria manifests in the lung; (ii) whether repetitive antigen encounter improves mycobacterial control; and (iii) how airway-resident CD8^+^ T cells differ from those embedded in the lung parenchyma, in their capacity to restrict mycobacterial growth. While CD4^+^ T cell help is a major contributor to the development of CD8^+^ T cell responses in chronic infection (*16*), it has previously been shown that airway luminal CD4^+^ T cells do not contribute to the anti-TB effect of murine airway CD8^+^ T cells (*17*). Of interest, mucosal BCG vaccination induces memory T cell subsets that are functionally superior (*7*), and evidence from other infection models suggests that secondary (2^0^) memory T cells exhibit superior lung accumulation and protective capacity (*18*). In TB, however, BCG revaccination, has not consistently improved protection in humans (*19*), despite evidence that repeated mycobacterial exposure can enhance TB control in animal models (*20*, *21*). Therefore, reinfection studies warrant further investigation as the functional differences in primary (1^0^) and 2^0^ memory T cell responses in human TB remain underexplored.

Here, we compared 1^0^ and 2^0^ CD8^+^ memory T cell responses following mucosal BCG exposure, focusing on lung and bronchoalveolar lavage fluid (BALF). Using an extended infection timeline, we examined whether repeated antigen encounter enhances polyclonal memory CD8^+^ T cell durability and effector function. We show that, paradoxically, reduced numbers of pulmonary T_EM_ during a 2^0^ immune response correlate with accelerated mycobacterial clearance. This is explained by enhanced migration of airway-derived CD8^+^ T_EM_ to the spleen in parallel to the translocation of both BCG and *Mtb* to the spleen by migratory APC. Killing of bacteria in the spleen, could be further enhanced by adoptive transfer of a threshold number of airway-derived 2^0^ CD8^+^ T_EM._ These findings uncover a previously unknown immunological detour pathway, which enhances mycobacterial killing in the spleen, and reshape current paradigms of TB immunity.

## RESULTS

### 2^0^ response to BCG is characterized by a reduction in pulmonary CD8^+^ memory T cells

**Fig. 1.**
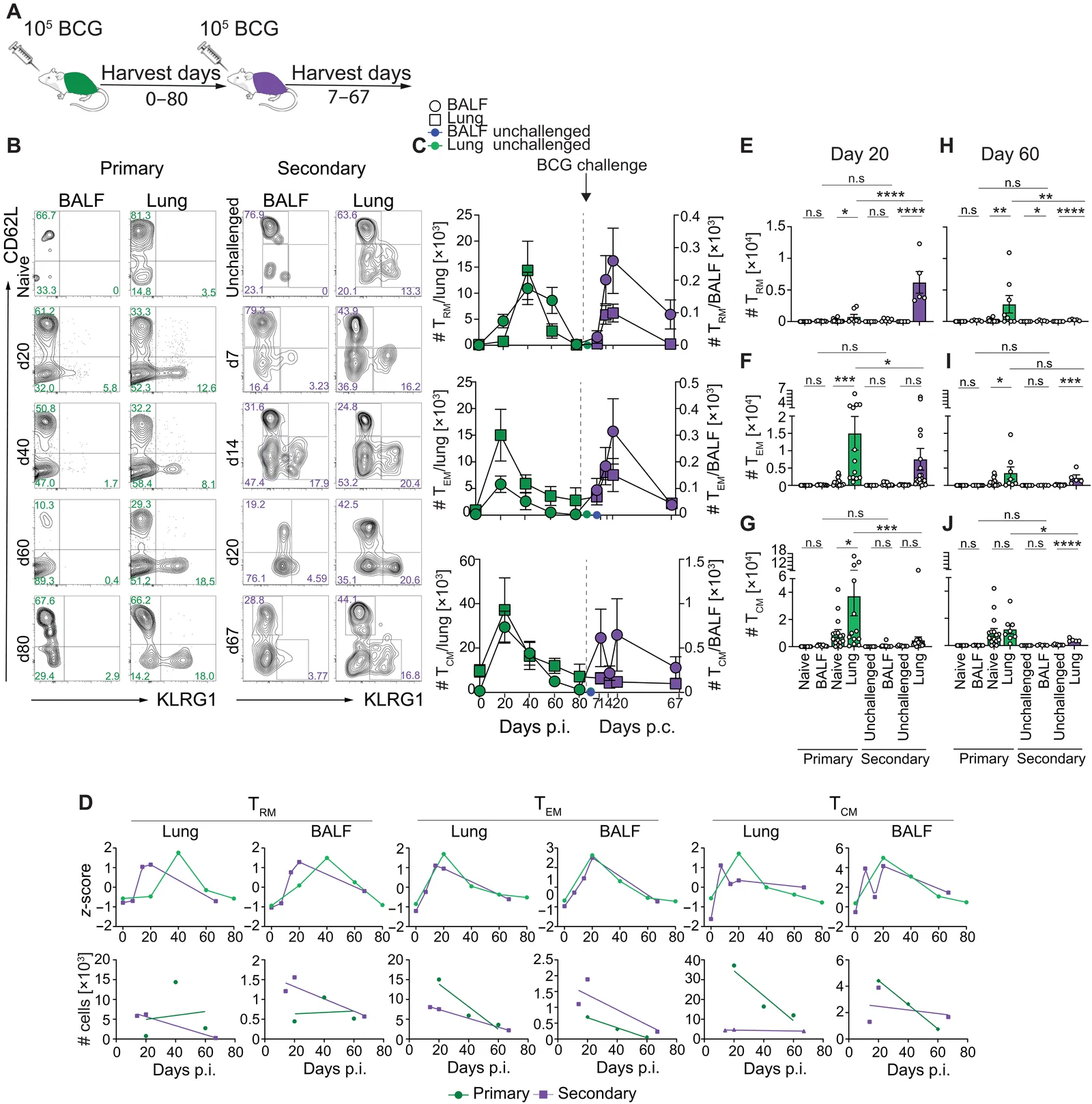
2^0^ response to BCG is characterized by a reduction in pulmonary CD8^+^ memory T cells. (**A**) Schematic of experimental plan. C57BL/6 mice were infected intratracheally (i.t.) with 10^5^ BCG (1^0^ infection in green) or left uninfected (naïve). Some mice were challenged i.t. with 10^5^ BCG 80 days after infection (2^0^ infection in purple) or left unchallenged. T_RM_, T_EM_, and T_CM_ were enumerated at various time points in the BALF (circle) and lung parenchyma (square) using flow cytometric analysis. (**B** and **C**) Representative flow plots (B) and absolute number (C) of T_RM_, T_EM_, and T_CM_ over time (BALF cell number plotted on the right *y* axis and lung cell number plotted on the left *y* axis). (**D**) Best-fit spline curves showing *z*-score (top) and linear (bottom) T_RM_, T_EM_, and T_CM_ numbers in BALF and lung over time during 1^0^ (green) and 2^0^ (purple) infection. (**E** to **J**) Comparison of CD8^+^ T_RM_ (top), T_EM_ (center), and T_CM_ (bottom) cell numbers in BALF and lung at 20 and 60 days after 1^0^ and 2^0^ infection. Statistics shown for BALF 1^0^ versus 2^0^; lung 1^0^ versus 2^0^; BALF and lung compared to naïve; BALF and lung compared to unchallenged. Results are presented as data means (D), data means ± SEM (C), individual data points ± SEM (E to J), and representative flow plots (B) from at least two pooled independent experiments (*n* = 3 to 16 mice per group). Statistical analyses: One-way ANOVA followed by Dunnett’s multiple comparison test; significant differences are indicated by asterisks: *P < 0.05, **P < 0.01, ***P < 0.001, ****P < 0.0001. p.i., postinfection; p.c., postchallenge. Illustration (A) was generated with NIAID NIH BioArt Source images under Public Domain (bioart.niaid.nih.gov).

By definition, a 2^0^ immune response to a foreign antigen or pathogen should be faster and larger in magnitude and lead to quicker pathogen clearance compared to a 1^0^ response (*22*). To test the relevance of this paradigm in mycobacterial infection, we first examined the mucosal response of various CD8^+^ memory T cell subsets to BCG. BCG was administered intratracheally (i.t.) to simulate the effect of bacterial infection on airway mucosal immunity (Fig. 1A) (*7*). C57BL/6 mice were administered 10^5^ colony-forming units (CFU) of BCG SSI i.t. (1^0^ infection; green) and were euthanized on day 0, 20, 40, 60, or 80 postinfection (p.i.) (Fig. 1A). Some mice received a BCG dose again on day 80 p.i. (2^0^ infection; purple) and were euthanized at 7, 14, 20, and 67 days postchallenge (p.c.). BALF, lung, spleen, and mediastinal lymph node (MLN) were harvested, and cells were isolated and analyzed by flow cytometry. Effector versus memory CD8^+^ T cells were distinguished based on phenotypic markers as shown (Table 1) (*23*, *24*). The expansion, contraction, and establishment of long-lived memory was tracked during both 1^0^ and 2^0^ responses, proportionally (Fig. 1B and fig. S1, A to D) and numerically (Fig. 1C). During the 1^0^ response to BCG, T_CM_ and T_EM_ expanded and peaked on day 20 in the BALF and lung while T_RM_ peaked on day 40 (Fig. 1C, green). This was followed by a contraction phase, marked by exhaustion and replicative senescence, returning to baseline levels on day 80 p.i. (*25*). Unusually, the number of CD8^+^ memory T cells during the 2^0^ response was lower or the same as the 1^0^, with T_EM_ and T_CM_ not reaching a peak of similar magnitude on day 20 p.c. (Fig. 1C, purple). Only T_RM_ expanded somewhat faster, reaching a peak comparable to that of the 1^0^ response by day 20 p.c. When we calculated and plotted the *z*-score of memory CD8^+^ T cell numbers over time, it was evident that the 2^0^ CD8^+^ T_EM_ and T_CM_ response in the BALF and lung were neither faster nor larger than the 1^0^ response (Fig. 1D, top). Specifically, the expansion and contraction profiles of T_EM_ and T_CM_ did not shift to the left, as predicted, along the infection timeline (Fig. 1D, top and bottom).

Memory CD8^+^ T cells have a higher antigen threshold for activation than naïve CD8^+^ T cells (*26*). However, *Mtb* phagosomes are secluded from the MHC class I processing pathway; therefore, antigen presentation by *Mtb*-infected macrophages is limited in the lung (*27*). In such a low-antigen environment, naïve T cells that express high-affinity T cell receptors (TCRs) are preferentially selected, dwarfing a memory recall response (*28*). As a result, memory T cells fail to proliferate and undergo attrition. On the basis of these reports, we compared the number of T_RM_, T_EM_, and T_CM_ subsets in the BALF and lung on day 20 of a 1^0^ and 2^0^ response with naïve mice (Fig. 1, E to G). All three subsets were significantly increased in number in the lung during 1^0^ response (green), whereas the increase in BALF was not significant. T_EM_ and T_CM_ numbers remained unchanged compared to the unchallenged control group during the 2^0^ response (purple), although T_RM_ numbers increased. Direct comparison of 1^0^ and 2^0^ responses paradoxically revealed a significant reduction in T_EM_ and T_CM_ numbers in the lung during the 2^0^ response (*P < 0.05, ***P < 0.001), and a significant increase in T_RM_ numbers (****P < 0.0001) (Fig. 1, E to G). This profile changed on day 60 p.i. (Fig. 1, H to J) wherein T_EM_ numbers during the 1^0^ and 2^0^ responses equalized (*P* = ns), and difference in T_CM_ numbers minimized (*P < 0.05). This shift appeared to result from a significant increase in T cell numbers during the 2^0^ response (****P < 0.0001), compared to the unchallenged controls. In summary, our data challenge the existing paradigm as T_EM_ and T_CM_ numbers during the 2^0^ response to BCG are reduced in the lung compared to 1^0^ in the early stage of infection, with numbers equalizing only in the late stage.

### Accelerated BCG clearance from BALF and lung during the 2^0^ response correlates with a reduction in CD8^+^ T cell numbers in the lung

**Fig. 2.**
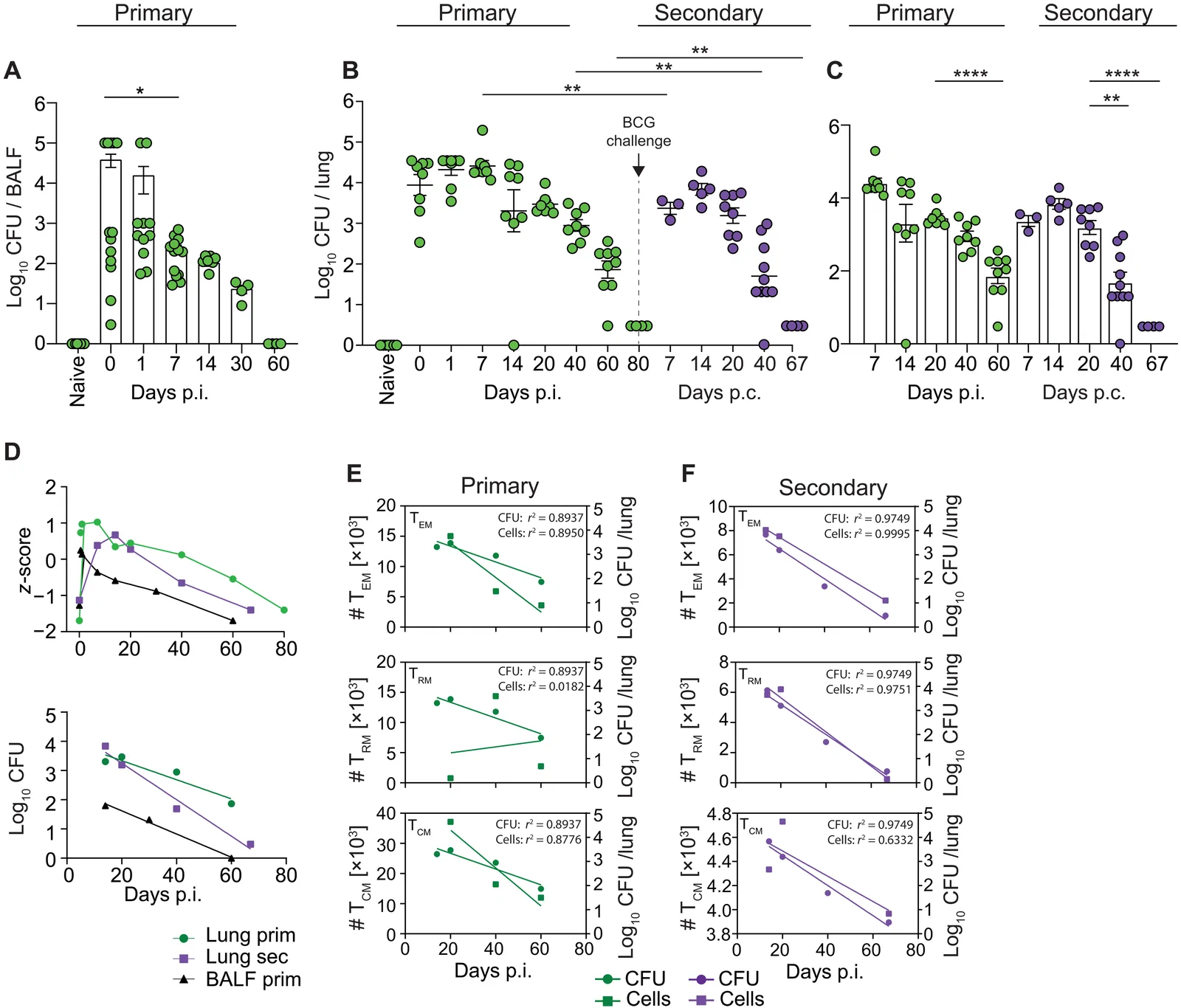
Accelerated BCG clearance from BALF and lung during the 2^0^ response correlates with a reduction in CD8^+^ T cell numbers in the lung. C57BL/6 mice were infected i.t. with 10^5^ BCG (1^0^ infection in green) or left uninfected (naïve) as shown in Fig. 1A. Some mice were challenged i.t. with 10^5^ BCG 80 days after infection (2^0^ infection in purple) or left unchallenged. (**A** to **C**) Number of BCG bacteria in the BALF (A) and lung parenchyma (B and C) during 1^0^ (green circles) and 2^0^ (purple circles) infection, enumerated over time. (**D**) Best-fit spline curves showing *z*-score (top) and log-transformed (bottom) CFU in BALF and lung over time during 1^0^ (green, black) and 2^0^ (purple) infection. (**E** and **F**) Linear regression of CFU and T_RM_ or T_EM_ or T_CM_ numbers in lung over time during 1^0^ (E, green) and 2^0^ (F, purple) infection. Results are presented as data means (D to F) and individual data points ± SEM (A to C) from at least two pooled independent experiments (*n* = 4 to 10 mice per group). Statistical analyses: One-way ANOVA followed by Dunnett’s multiple comparison test; significant differences are indicated by asterisks: *P < 0.05, **P < 0.01, ***P < 0.001, ****P < 0.0001. p.i., postinfection; p.c., postchallenge.

We also enumerated the BCG bacterial load in the BALF and lung, expressed as CFU, on agar. C57BL/6 mice were infected with a first and second dose of 10^5^ CFU of BCG i.t. (1^0^ and 2^0^ infection respectively) as shown in Fig. 1A. BALF, lung, spleen, and MLN were harvested. Expectedly, BCG CFU levels were high in the BALF on day 0 and 1 p.i. compared to naïve mice (Fig. 2A). A sharp reduction in CFU was observed on day 7 p.i. that remained consistently low on days 14 and 30, eventually reaching baseline levels on day 60 p.i., as in naïve mice.

In the lung, BCG CFU levels (Fig. 2B) were high on day 0, 1, 7, 14, and 20 p.i. during both 1^0^ (green) and 2^0^ (purple) infection (Fig. 2B). Comparison of successive 1^0^ infection time points indicated that there was minimal change in bacterial load (*P* = ns) (Fig. 2C, green). There was no significant reduction in CFU between day 20 and 40 as well as between day 40 and 60 p.i. (Fig. 2C, green). In contrast, CFU clearance during the 2^0^ infection was significantly accelerated as compared to the 1^0^ infection (Fig. 2C, purple). A notable 1.5 log reduction in bacterial load occurred during the 2^0^ infection between day 20 and 40 p.c. (**P < 0.01), relative to 0.5 log reduction during 1^0^ (*P* = ns). Further, a 2.7 log reduction in bacterial load occurred during the 2^0^ infection between day 20 and 60 p.c., relative to 1.6 log reduction during 1^0^, with CFU reaching baseline levels on day 67 p.c. Together, these results demonstrate that BCG gradually decreases over approximately 40 days (between day 20 and 60 p.i.) during a 1^0^ infection, whereas the reduction is accelerated to approximately 20 days (between day 20 and 40 p.c.) during a 2^0^ infection. Curiously, accelerated bacterial clearance during a 2^0^ infection correlated with a reduction in memory T cell numbers in the lung (Fig. 2, D to F, and fig. S2), prompting us to further investigate this unexpected result.

### BCG reduction in the spleen corresponds to CD8^+^ T_EM_ migration from the BALF and lung

**Fig. 3.**
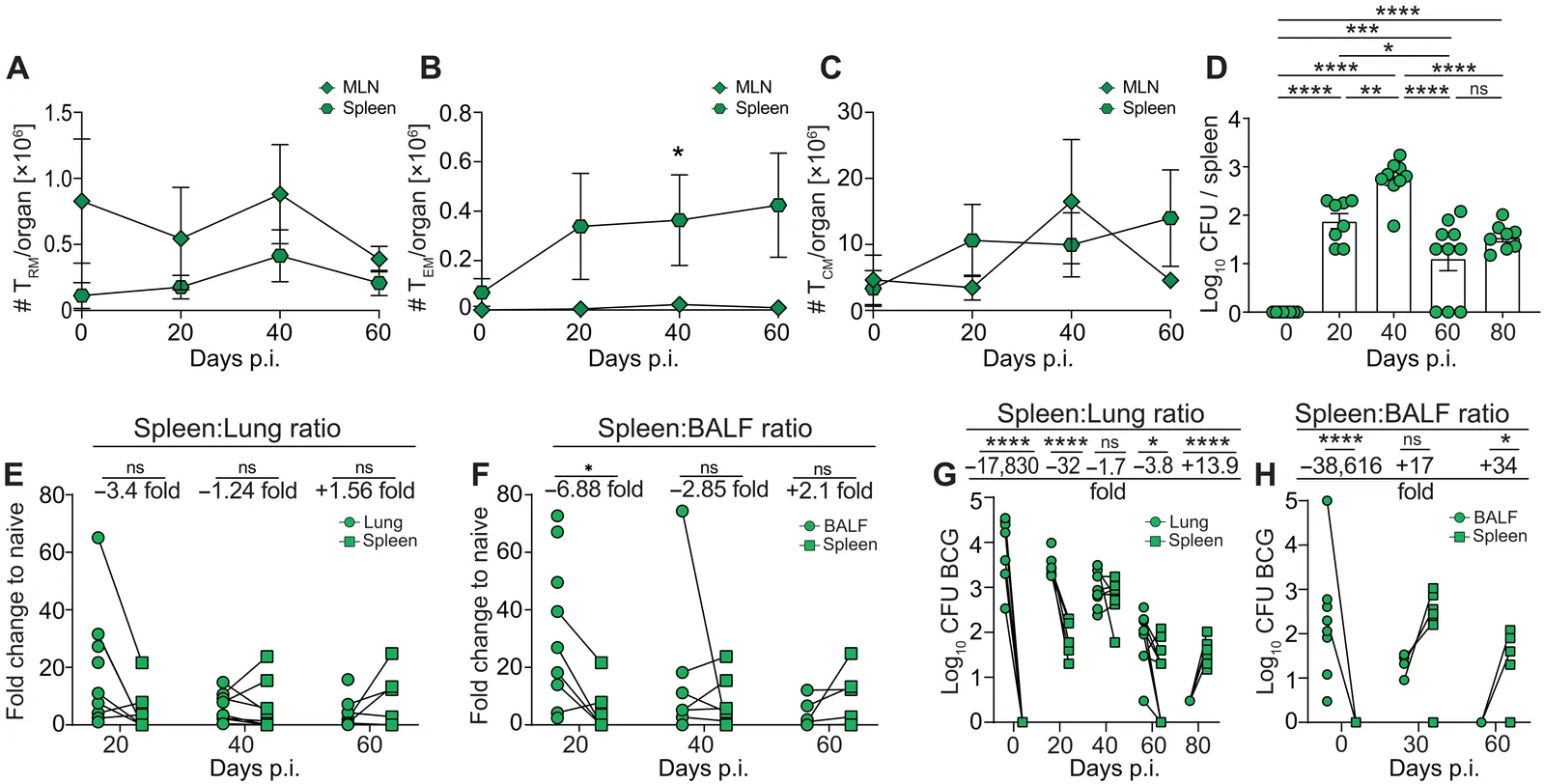
BCG reduction in the spleen corresponds to CD8^+^ T_EM_ migration from the BALF and lung. (**A** to **C**) T_RM_, T_EM_, and T_CM_ were enumerated at various time points in the MLN (diamond) and spleen (hexagon) using flow cytometric analysis. (**D**) The number of BCG bacteria in the spleen over time. (**E** and **F**) Fold change of T_EM_ numbers in spleen versus lung (E), and spleen versus BALF (F) at 20, 40, and 60 days p.i. (**G** and **H**) Fold change in BCG CFU in spleen versus lung (G) and spleen versus BALF (H) over time. Results are presented as individual data points (E to H), individual data points ± SEM (D), and data means ± SEM (A to C) from two pooled independent experiments (*n* = 5 to 10 mice per group). Statistical analyses: One-way ANOVA followed by Dunnett’s multiple comparison test (A to D) or Mann-Whitney test (E to H); significant differences are indicated by asterisks: *P < 0.05, **P < 0.01, ***P < 0.001, ****P < 0.0001. p.i., postinfection.

Delayed activation of *Mtb*-specific CD4^+^ T cells is attributed to the bacteria being confined to a compartment in the lung that does not support effective antigen presentation (*13*). This sequestration allows for significant expansion of *Mtb* in the lung. The earliest detectable immune responses are observed 2 to 3 weeks p.i. when live bacteria are translocated by DC to the MLN (*13*, *14*). On the basis of these reports, we tested whether the reduction in both BCG CFU and CD8^+^ T_EM_ (hereafter T_EM_) in the BALF and lung is due to their migration to the spleen (lack of CD62L excludes T_EM_ migration to MLN). Mice were infected with BCG as depicted in Fig. 1A. Contrary to the memory T cell profile in the BALF and lung (Fig. 1), CD8^+^ T_CM_ and T_RM_ numbers remained unchanged during the 1^0^ response in the spleen and MLN, compared to naïve mice (day 0 p.i.) (Fig. 3, A to C). However, T_EM_ numbers increased significantly in the spleen over time, particularly on day 40 p.i. (*P* < 0.05). This increase in T_EM_ number corresponded to a significant increase in spleen CFU on day 20 and 40 p.i. relative to naïve mice (day 0 p.i.) (****P < 0.0001) (Fig. 3D) and an average 0.9 log increase in bacterial load between day 20 and 40 p.i. (**P < 0.01).

Next, the BALF, lung, and spleen T_EM_ values were normalized as fold change to naïve control mice. Spleen:lung and spleen:BALF T_EM_ ratios were assessed (Fig. 3, E and F). Both ratios slightly increased over the timeline of day 20, 40, and 60 p.i., shifting from negative to positive values. The spleen:lung BCG CFU ratio also gradually increased over the timeline of day 0, 20, 40, 60, and 80 p.i. (Fig. 3G) and the spleen:BALF CFU ratio followed a similar trajectory over day 0, 30, and 60 p.i. (Fig. 3H). Both the CFU ratios shifted from negative to positive values. On day 60 and 80 p.i., BALF, lung, and spleen CFU reduced on an average by 0.5 to 1 log, compared to the previous time point, and began to reach baseline levels, indicative of bacterial killing. Collectively, these data suggest that T_EM_ may be migrating from the lung to the spleen on day 20 p.i. when BALF and lung T_EM_ numbers are very high. In the early stage, the spleen:BALF and spleen:lung ratios are therefore negative (<0). As T_EM_ continue to migrate, BALF and lung T_EM_ numbers equalize with the spleen and then decrease relative to the spleen, resulting in a positive ratio (>0). Bacterial translocation from the BALF or lung to the spleen follows a similar pattern [ratio starts as a very negative value (<−1000), then equalizes and eventually increases to >1]. Together, a relationship emerged between T_EM_ and CFU in the airways, lung, and spleen. An increase (or decrease) in CFU was paralleled by an increase (or decrease) in T_EM_, consistent with reports of CD4^+^ T cells and *Mtb* migrating to MLN to find antigen (*13*). We rationalized that if BCG clearance is mediated by spleen T_EM_, then a therapeutic strategy that increases T_EM_ numbers in the spleen may prove beneficial.

### 2^0^ T_EM_ migrate from the lung to the spleen for antigen signaling

**Fig. 4.**
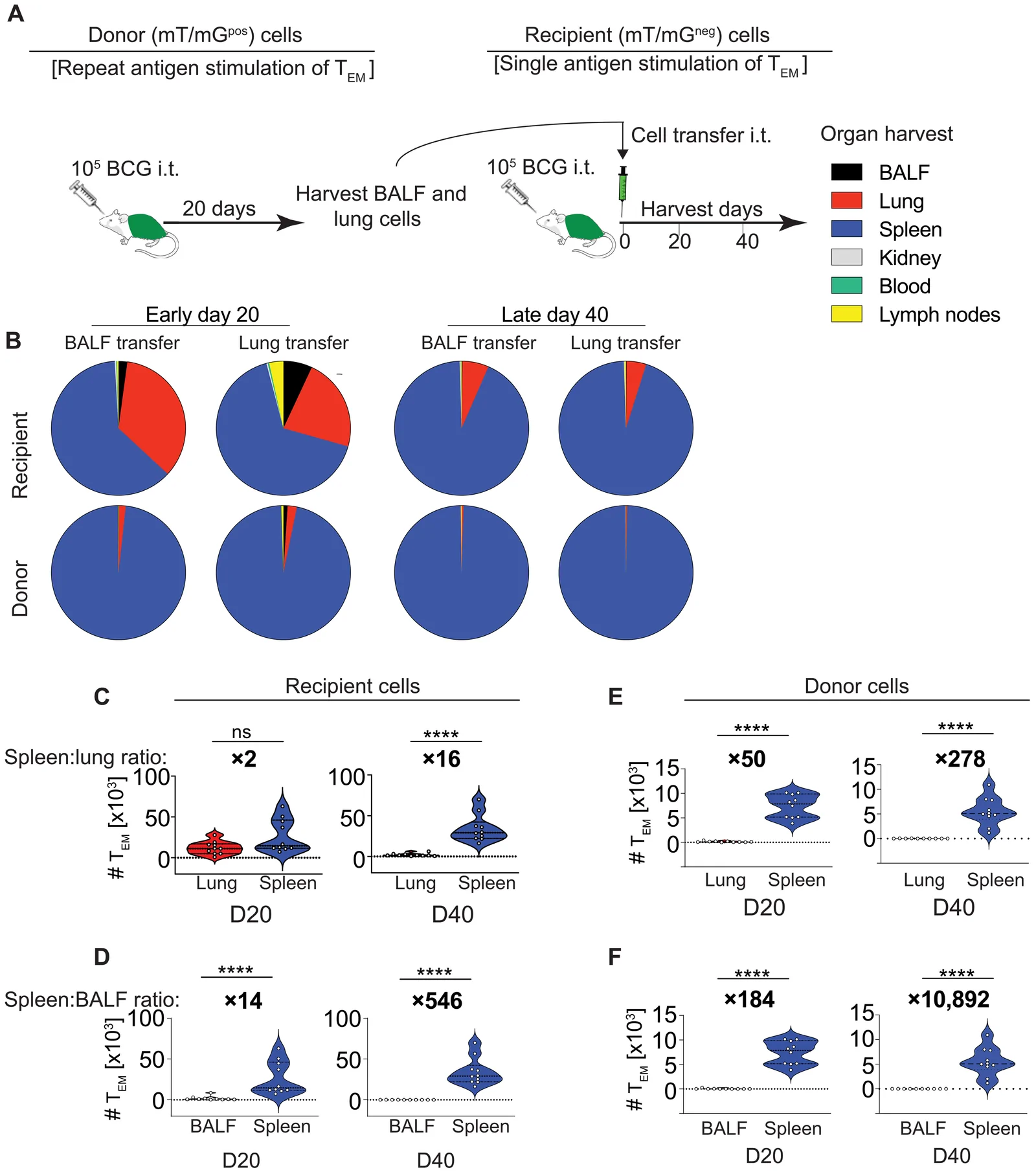
2^0^ T_EM_ migrate from the lung to the spleen for antigen signaling. (**A**) Experimental scheme for T cell trafficking analyses. mT/mG mice were infected i.t. with 10^5^ BCG and cells were isolated from BALF and lung 20 days p.i. BALF and lung cells were adoptively transferred into C57BL/6 recipient mice via the i.t. route. Recipient mice were infected i.t. with 10^5^ BCG at the same time as cell transfer. (**B**) Localization of the mT/mG^pos^ and mT/mG^neg^ cells in various organs (BALF, lung, spleen, kidney, blood, and lymph nodes) was assessed on day 20 (early) and day 40 (late) after cell transfer. (**C** to **F**) Fold change in spleen versus lung and spleen versus BALF cells in mT/mG^neg^ (C and D) and mT/mG^pos^ population (E and F) on day 20 and day 40 after cell transfer. Results are presented as individual data points ± SEM (C to F) and pie charts (B) from two pooled independent experiments (*n* = 5 to 10 mice per group). Statistical analyses: Mann-Whitney test; significant differences are indicated by asterisks: ****P < 0.0001. Illustration (A) was generated with NIAID NIH BioArt Source images under Public Domain (bioart.niaid.nih.gov).

In the next set of experiments, we investigated whether the significant reduction in 2^0^ T_EM_ numbers (Fig. 1F, purple) was due to their faster migration from the lung to the spleen following a second antigen encounter. To address this, mT/mG mice were used as a tool for tracking T_EM_. Donor mT/mG mice were infected with BCG, and 20 days later, BALF and lung cells were harvested (Fig. 4A). BALF and lung cells were adoptively transferred into recipient mice via the intratracheal route (Fig. 4A). Recipient mice were infected with BCG, and the localization of T_EM_ was tracked in various organs (BALF, lung, spleen, kidney, blood, and lymph nodes) on day 20 and 40 p.i. These experiments were intended to compare 1^0^ T_EM_ migration before [mT/mG^neg^] and after [mT/mG^pos^] a second antigen encounter. We have previously reported that CD8^+^ T cells exhibit directed migration toward infected macrophages in peripheral tissues (*29*). Accordingly, a large number of mT/mG^neg^ T_EM_ had localized in the lung (red) and BALF (black) on day 20 p.i. (Fig. 4B, top) that coincided with BCG-infected alveolar macrophages (AM) also localizing within the BALF and lung (*30*). The distribution profile changed significantly on day 40 p.i., with most of the T_EM_ localizing to the spleen (blue). In contrast, repeat antigen-stimulated mT/mG^pos^ T_EM_ were almost entirely absent from the BALF and lung on day 20 p.i., with most having localized already in the spleen, a profile that remained unchanged on day 40 (Fig. 4B, bottom). Thus, 2^0^ T_EM_ migrated from the BALF and lung to the spleen at least 20 days earlier compared to 1^0^ T_EM_.

Both mT/mG^pos^ and mT/mG^neg^ T_EM_ decreased significantly in the BALF (black bars) and lung (red bars) on day 40 p.i. compared to day 20 (fig. S3, C to F). These differences were less evident or nonexistent in the spleen (fig. S3, C to F, blue bars, and G). For ease of interpretation, data from mice that received either BALF or lung cells were pooled (Fig. 4, C to F). The spleen:lung ratio of mT/mG^pos^ T_EM_ increased (50- and 278-fold) (Fig. 4E) compared to mT/mG^neg^ T_EM_ (2- and 16-fold) (Fig. 4C). Similarly, the spleen:BALF ratio of mT/mG^pos^ T_EM_ increased (184- and 10,892-fold) (Fig. 4F) compared to mT/mG^neg^ T_EM_ (14- and 546-fold) (Fig. 4D) on day 20 and 40 p.i., respectively. The 16-fold increase in spleen:lung ratio on day 40 p.i. by mT/mG^neg^ cells (Fig. 4C, right) was reached as early as day 20 p.i. by mT/mG^pos^ cells (Fig. 4E, left). Next, mT/mG^neg^ recipient mice (Fig. 4, C and D) were compared to BCG-infected wild-type mice. The spleen:lung and spleen:BALF positive ratios of +1.56- and +2.1-fold, respectively, were not reached in the wild-type mice until day 60 p.i. (Fig. 3, E and F). This indicates that mT/mG^pos^ cells may have a paracrine effect in potentiating mT/mG^neg^ cell function via T cell cytokines. These data show that a second pulmonary encounter with antigen significantly enhanced the migration capacity of mT/mG^pos^ T_EM_ toward the spleen, migrating faster to the spleen by approximately 20 days compared to mT/mG^neg^ T_EM_.

### Increased number of 2^0^ T_EM_ show specificity for mycobacterial antigen

**Fig. 5.**
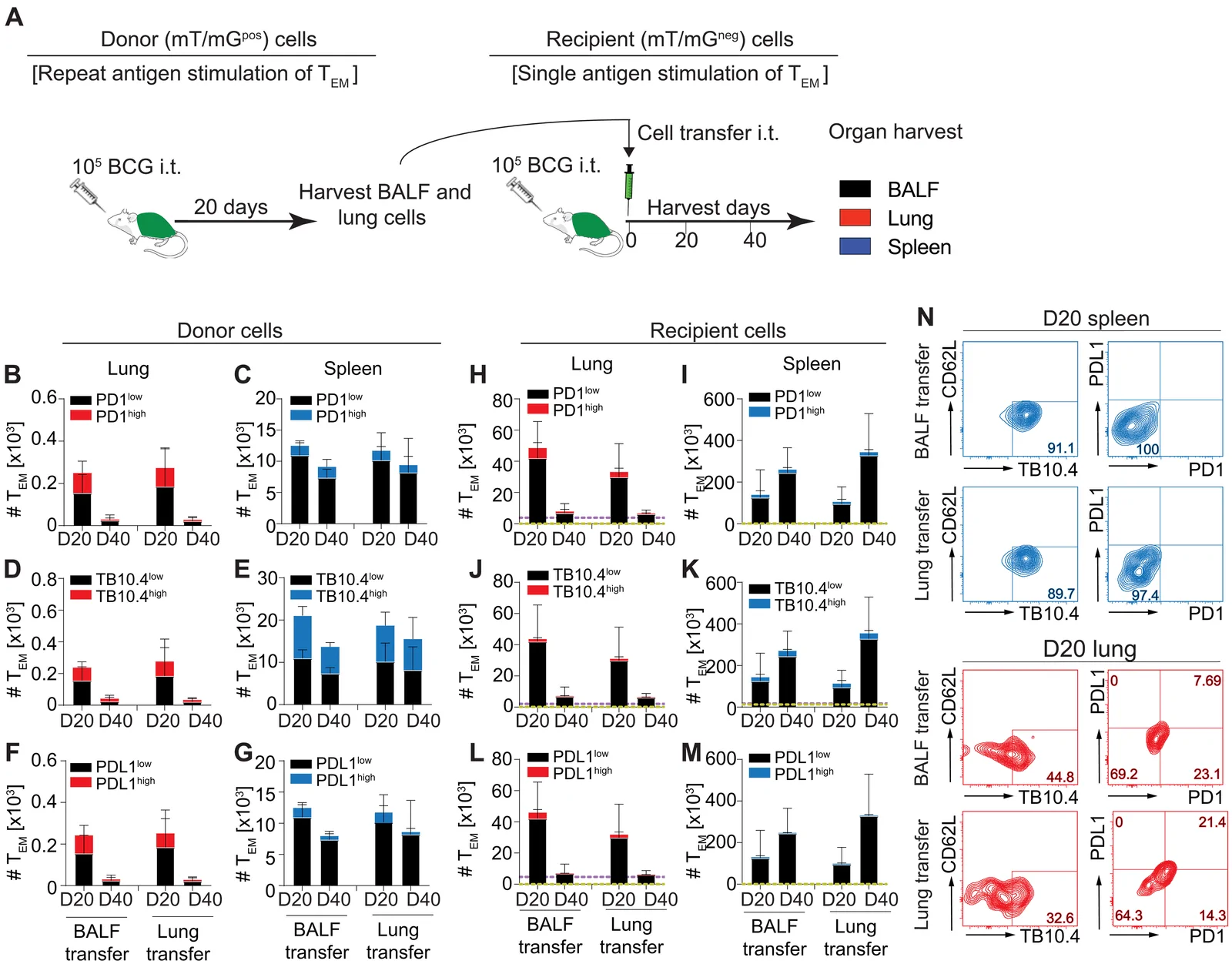
Increased number of 2^0^ T_EM_ show specificity for mycobacterial antigen. (**A**) Experimental scheme for T cell trafficking analyses as described in Fig. 4A. (**B** and **C**) Number of PD1^hi^ (red, blue) and PD1^lo^ (black) mT/mG^pos^ donor cells after repeat antigen stimulation in lung (B) and spleen (C). (**H** and **I**) mT/mG^neg^ recipient cells after single antigen stimulation in lung (H) and spleen (I). (**D** and **E**) Number of TB10.4^hi^ (red, blue) and TB10.4^lo^ (black) mT/mG^pos^ donor cells after repeat antigen stimulation in lung (D) and spleen (E). (**J** and **K**) mT/mG^neg^ recipient cells after single antigen stimulation in lung (J) and spleen (K). (**F** and **G**) Number of PDL1^hi^ (red, blue) and PDL1^lo^ (black) mT/mG^pos^ donor cells after repeat antigen stimulation in lung (F) and spleen (G). (**L** and **M**) mT/mG^neg^ recipient cells after single antigen stimulation in lung (L) and spleen (M). Baseline levels in no cell control mice are shown as pink (day 20) and yellow lines (day 40). (**N**) PD1 and PDL1 expression on TB10.4^+^ cells in the spleen and lung on day 20 following cell transfer. Results are presented as mean ± SEM (B to M) and representative FACS plots (N) from two pooled independent experiments (*n* = 5 to 10 mice per group). Statistical differences for (B) to (M) are shown in fig. S4 (A and B). Illustration (A) was generated with NIAID NIH BioArt Source images under Public Domain (bioart.niaid.nih.gov).

Mycobacterial antigen Ag85B transgenic CD4^+^ T cells exhibit substantial proliferation at 28 days and thereafter contract 10-fold due to limited antigen availability (*31*). ESAT-6 transgenic CD4^+^ T cells on the other hand proliferate continuously beyond 28 days but become exhausted due to chronic antigen stimulation. We next tested (Fig. 5A) whether the protective capacity of CD8^+^ T_EM_ is restricted by the same factors in the lung that restrict CD4^+^ T cells (*31*). mT/mG^pos^ and mT/mG^neg^ cells were tested for expression markers of antigenic signaling (Fig. 5, B to M, and fig. S4, A to H). mT/mG^neg^ T_EM_ in the lung, spleen (Fig. 5, H to M), and BALF (fig. S4, D, F, and H) were mostly PD1^lo^, TB10.4^lo^, and PDL1^lo^, indicating that these cells were exposed to limited antigenic signaling. In contrast, approximately 40% of the mT/mG^pos^ T_EM_ in the lung were PD1^hi^, TB10.4^hi^, and PDL1^hi^, expectedly due to repeated antigen encounter first in the donor and then the recipient (Fig. 5, B, D, and F). mT/mG^pos^ and mT/mG^neg^ T_EM_ numbers in the donor and recipient respectively decreased on day 40 p.i. as above. mT/mG^neg^ T_EM_ numbers reached no cell control levels (dotted line) on day 40 p.i., suggesting that like the Ag85B-transgenic CD4^+^ T cell response, antigen may be a limiting factor here. There was no indication that T_EM_ were functionally exhausted, rather they had acquired better migration capability (Fig. 5, B to G).

We subsequently tested differential antigenic signaling in lung and spleen following adoptive transfer of BALF and lung cells (fig. S4, A and B). A similar number of PD1^hi^ and PDL1^hi^ T_EM_ were detected in the lung and spleen on day 20 p.i. in the mT/mG^neg^ population (*P* = ns) (Fig. 5, H to M, and fig. S4B), indicating there was no differential signaling. In contrast, there was significant exposure to antigenic signaling in the mT/mG^pos^ T_EM_ population in the spleen compared to lung as evident from PD1^hi^ (**P < 0.01) and PDL1^hi^ (**P < 0.01) cell numbers on day 20 p.i. (Fig. 5, B to G, and fig. S4A). An increased recognition of TB10.4 tetramer by spleen T_EM_ (****P < 0.0001) suggested that they had significant exposure to mycobacterial antigen (Fig. 5E). TB10.4^hi^ cells were less differentiated (PD1^lo^) in the spleen on day 20 p.i. (Fig. 5N). In this context, CD8^+^ memory T cells express limited PD1 following heterologous prime boost vaccination with vesicular stomatitis virus (*32*).

mT/mG^pos^ BALF T_EM_ (fig. S4, C, E, and G) contracted faster, as early as day 20 p.i. This may be because BALF cells were more activated than lung cells on day 20 and 40 p.i. (fig. S4A). mT/mG^pos^ BALF cells expressed significantly higher PD1 (*P < 0.05), PDL1 (**P < 0.01), and TB10.4 antigen (****P < 0.0001), as compared to lung cells (*P* = ns, *P < 0.05) on day 40 p.i., differences that were also detected in the mT/mG^neg^ population (fig. S4, A and B). In comparison to the transfer of antigen-experienced cells, adoptive transfer of naïve tomato cells showed similar organ distribution to mT/mG^neg^ recipient cells 40 days after cell transfer, regardless of whether they encountered antigen (fig. S4, I to Q), suggesting that transfer of naïve cells cannot replicate the functional effects of antigen-experienced cells. Here, a second BCG exposure led to a significant increase in transferred mT/mG^pos^ cells in the lung (fig. S4K) and an increase in antigen-specific T_EM_ in the spleen (fig. S4M, blue bars). Together, a second antigen encounter enhanced migration of T_EM_ from the lung to the spleen and increased their specificity for mycobacterial antigen, potentially also because of the tissue microenvironment and/or trained immunity. These observations are consistent with other infection models (*33*–*35*). Further, airway T_EM_ responded better to antigen than lung T_EM_ in adoptively transferred mice, trafficking faster to the lung by at least 40 days, compared to control mice.

### 2^0^ T_EM_ accelerate mycobacterial translocation from the lung to the spleen

**Fig. 6.**
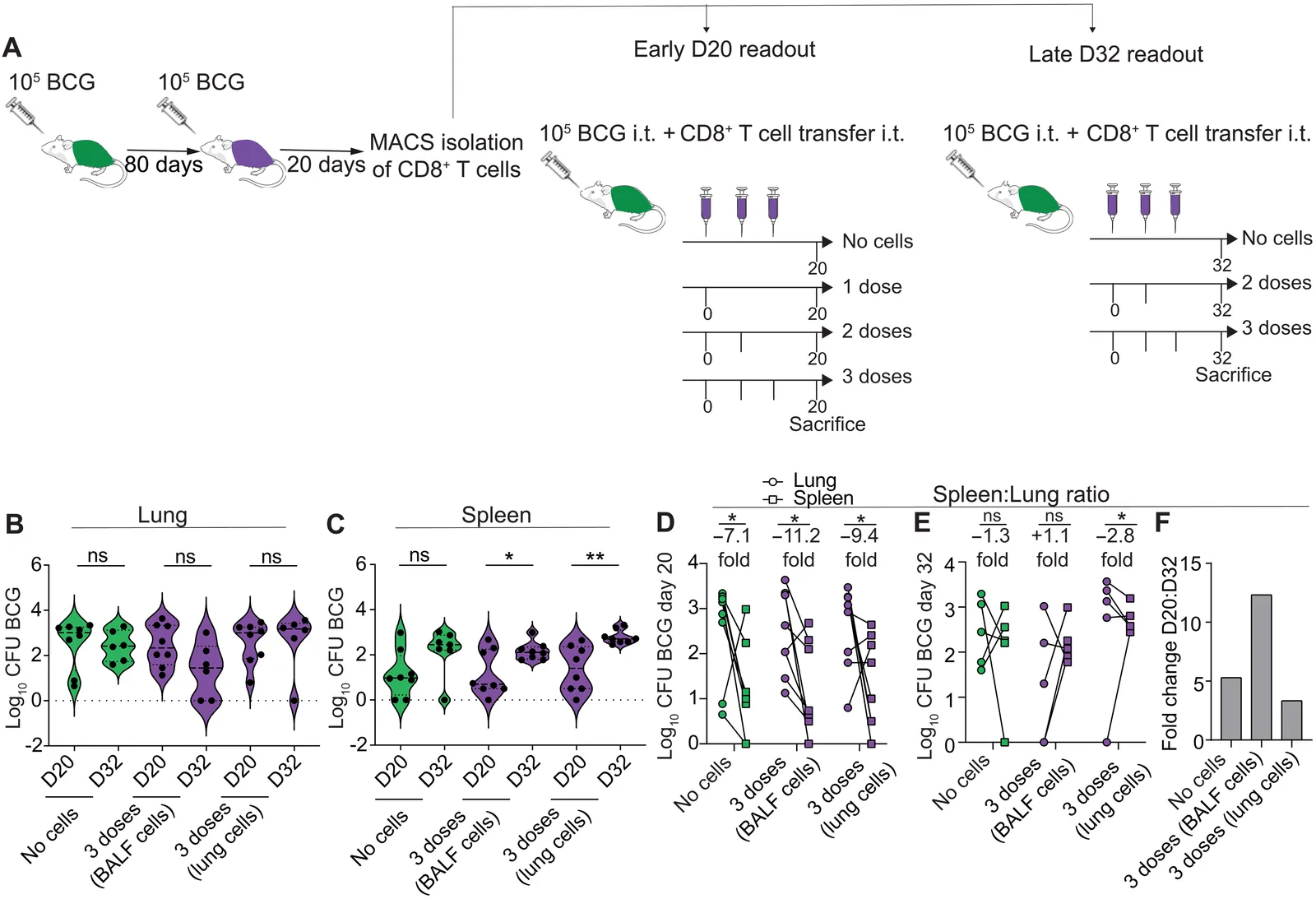
2^0^ T_EM_ accelerate mycobacterial translocation from the lung to the spleen. (**A**) Schematic of experiment. C57BL/6 donor mice were infected i.t. with 10^5^ BCG on day 0 (green). Mice were challenged i.t. with 10^5^ BCG 80 days after infection (purple). CD8^+^ T cells were isolated by MACS from BALF and lung in three batches 20 days after challenge and transferred i.t. into C57BL/6 recipients (green). Recipients were subsequently infected i.t. with 10^5^ BCG. Recipient mice received either one, two, or three doses of 10^5^ T_EM_ at early or late time points as indicated. Mice receiving no cells served as controls. (**B** and **C**) Comparison of CFU on day 20 versus day 32 in lung (B) and spleen (C). (**D** and **E**) Fold change in CFU in spleen versus lung on day 20 (D) and day 32 (E). (**F**) Fold change in CFU between day 20 and day 32 in spleen versus lung. Results are presented as individual data points in violin plots (B and C), individual data points (D and E), and fold changes (F) from at least two pooled independent experiments (*n* = 6 to 8 mice per group). Statistical analyses: One-way ANOVA followed by Dunnett’s multiple comparison test (B and C) or Mann-Whitney test (D and E); significant differences are indicated by asterisks: *P < 0.05, **P < 0.01, ***P < 0.001, ****P < 0.0001. Illustration (A) was generated with NIAID NIH BioArt Source images under Public Domain (bioart.niaid.nih.gov).

Because a correlative relationship had emerged in our above experiments between T_EM_ and CFU in the airways, lung, and spleen, we next tested whether T_EM_ directly regulate bacterial translocation from the lung to the spleen. To test this, C57BL/6 donor mice were administered 10^5^ BCG i.t. on day 0 and then again on day 80 p.i. Mice were euthanized 20 days after the second BCG infection (Fig. 6A). BALF and lung cells were harvested. CD8^+^ T cells were sorted by MACS. CD8^+^ T cells isolated from BALF and lung by MACS were enriched for CD8^+^ T_EM_ compared to presort analysis (fig. S3, A and B). They were CD44^+^CD62L^−^KLRG1^+^CD103^−^CD127^−^CD43^+^T-bet^+^CD69^+^ (fig. S6A) (Table 1) (*7*, *8*, *36*–*39*). Loss of CD62L and up-regulation of CD44 and CD69 unequivocally identified them as T_EM_ cells (*9*). They were clearly distinguishable from effector cells, which are CD127^int^KLRG1^lo^ (*9*, *36*). They were similar to short-lived effector cells, which bear the markers CD127^−^T-bet^+^KLRG1^+^ and were distinguishable from memory precursor effector cells (MPECs) that are CD127^+^T-bet^lo^KLRG1^−^ (*36*, *38*, *39*) (fig. S6A and Table 1).

BCG-infected recipient mice were adoptively transferred with either one, two, or three doses of 10^5^ MACS-purified CD8^+^ T cells as shown in Fig. 6A. Control mice received only BCG (no cells). CFU were assessed in relation to five parameters—time period (early versus late); organ (lung versus spleen); adoptive cell transfer (BALF cells versus lung cells); dosage (one, two, or three doses); and antigen encounter (single versus repeated encounter). We first confirmed previous results (*7*) that adoptive transfer of total BALF cells can lead to a reduction in lung CFU (fig. S5, A and B). One dose of MACS-sorted 10^5^ BALF CD8^+^ T cells did not decrease the bacterial load in the lung in recipient mice (*P* = ns), as compared to 0 doses, regardless of the day of transfer (days 1, 7, 14, and 20) (fig. S5, C and D). Similarly, increasing T cell numbers on day 1 or 14 had no effect (fig. S5E). In line with previously published reports (*17*), transfer of the CD4^+^ T cell–enriched CD8-negative BALF fraction did not reduce lung bacterial burden. Transfer of two or three doses of BALF or lung CD8^+^ T cells (fig. S6B) in the early day 20 stage of infection did not significantly lower bacterial burden in the lung and spleen (*P* = ns), compared to no cell control (Fig. 6B and fig. S6, C and D). The lung inflammatory infiltrate remained unchanged between groups (fig. S6, E and F). A 3-week delay in the onset of CD4^+^ T cell responses has been attributed to the time required for uptake of *Mtb* by DC and its transportation to the MLN (*13*, *14*). Accordingly, spleen CFU was lower than lung CFU in the early stage, on day 20 p.i., regardless of the dosage (Fig. 6, B and C, and fig. S6, C and D). The spleen:lung ratio was <1 (−7.1-, −11.2-, and −9.4-fold) (Fig. 6D). Earlier, mT/mG^pos^ BALF and lung T_EM_ had shown increased efficacy in migrating from the lung to the spleen (Fig. 4, E and F). Therefore, we concluded that increased migration of mT/mG^pos^ T_EM_ from the lung to the spleen was not paralleled by increased bacterial translocation from the lung to the spleen, at least in the early stage.

However, a 1 log reduction in lung CFU in mice that received three doses of BALF CD8^+^ T cells was observed in the late stage, on day 32 p.i., compared to the early stage (Fig. 6B and fig. S6, C and G). No such decrease was observed in groups that received two doses of BALF cells or two or three doses of lung cells (fig. S6G). Notably, spleen CFU increased significantly in groups that received three doses of BALF or lung cells in the late stage (Fig. 6C and fig. S6, D and H) but not in the no cell control group. The lung inflammatory infiltrate remained unchanged between groups (fig. S6, I and J). Three doses of BALF cells increased bacterial translocation from the lung to the spleen by approximately 3-fold, shifting the spleen:lung ratio to a positive value of >1 (+1.1-fold), as compared to zero doses (−1.3-fold) or three doses of lung cells (−2.8-fold) (Fig. 6E). A similar threefold increase was detected in day 20:day 32 ratio (Fig. 6F). Of note, wild-type mice did not shift to a positive spleen:lung ratio until day 80 p.i. (Fig. 3G). Together, three doses of enriched BALF T_EM_ accelerated BCG translocation from the lung to the spleen threefold. However, translocation was not accompanied by immediate bacterial killing in the spleen, at least on day 32 p.i.; we reasoned that this will likely ensue at a later time point.

### Transfer of a threshold number of T_EM_ decreases AM numbers and recruits migratory APC

**Fig. 7.**
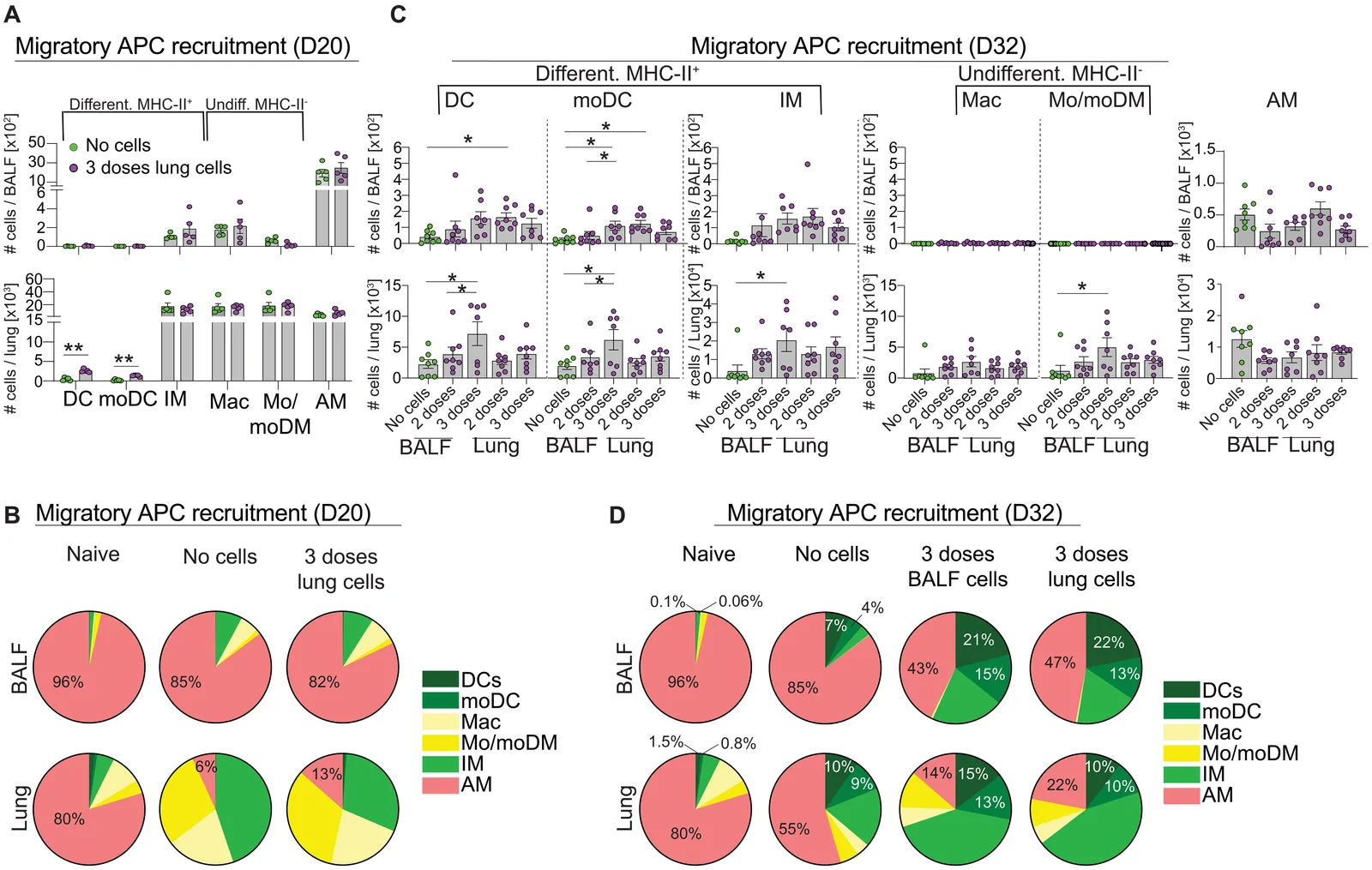
Transfer of a threshold number of T_EM_ decreases AM numbers and recruits migratory APC. (**A**) Total number of dendritic cells (DC), monocyte-derived DC (moDC), interstitial macrophages (IM), macrophages (Mac), monocyte-derived macrophages (moM), and alveolar macrophages (AM) in BALF and lung on day 20 after cell transfer. (**B**) Pie charts showing proportion of APC subsets in BALF and lung in naïve mice versus BCG-infected mice with or without cell transfer on day 20. (**C**) Total number of DC, moDC, IM, Mac, moDM, and AM in BALF and lung following transfer of two or three doses of BALF or lung cells or no cells on day 32. (**D**) Pie charts showing proportion of APC subsets in BALF and lung in naïve mice versus BCG-infected mice with or without cell transfer on day 32. Results are presented as individual data points ± SEM (A and C) and pie charts (B and D) from at least two pooled independent experiments (*n* = 6 to 8 mice per group). Statistical analyses: One-way ANOVA followed by Dunnett’s multiple comparison test; significant differences are indicated by asterisks: *P < 0.05, **P < 0.01.

Transport of mycobacteria from the lung to the lymphatic system depends on migratory APC (*13*, *14*). Hence, we also assessed whether adoptive transfer of T_EM_ is associated with a change in BALF and lung APC subsets. MHC-II expression was used as a marker to distinguish differentiated (MHC-II^+^) from undifferentiated (MHC-II^−^) APC (*40*), as mycobacteria-specific CD8^+^ T cells fail to recognize MHC-I^+^ lung APCs in infected mice (*41*). At 20 days p.i., adoptive transfer of three doses of lung cells had almost no effect on any of the APC types, including DC, monocyte-derived dendritic cells (moDC), macrophages (Mac), monocyte-derived macrophages (moDM), interstitial macrophages (IM), and AM, present in the BALF or lung, as compared to no cell control (Fig. 7A). The exception is very low DC and moDC numbers in the lung, which increased relative to the controls (**P < 0.01). Thus, by day 20 p.i., the APC population was largely composed of undifferentiated MHC-II^−^ monocytes/moDM or macrophages, aligning with the monocytosis observed in *Mtb*-infected mice at 2 and 4 weeks p.i. (*42*, *43*) (Fig. 7B). In addition, IM, which have a dual role in controlling bacterial growth while also being permissive to its replication (*44*), and AM, which promote nutritionally permissive bacterial growth, were also present in large numbers (*42*). DC and moDC numbers in the BALF and lung of no cell control mice increased even more on day 32 p.i., while IM, undifferentiated macrophages, and monocytes/moDM numbers significantly declined (fig. S6K). AM numbers slightly decreased in the BALF, possibly due to relocation from the airways to the lung interstitium (*30*).

The number of differentiated MHC-II^+^ migratory APC including DC, moDC, and IM significantly increased in the BALF and lung of mice that received three doses of BALF cells, as quantified by flow cytometry (*P* < 0.05) (Fig. 7, C and D). In addition, AM numbers dropped in the BALF from 505 cells (85%) in the no cell control to 320 cells (43%) in treated mice; similarly, numbers dropped in the lung from 12,562 cells (55%) in the control to 6725 cells (14%) in the treated mice (Fig. 7, C and D). Undifferentiated MHC-II^−^ Mac and moDM numbers were very low and mostly indistinguishable from no cell control. Three doses of lung cells were less effective in recruiting migratory APC (*P* = ns) (Fig. 7C); further confirmation was obtained by comparing zero doses with three doses of BALF and lung cells (fig. S6, L and M). Together, these results show that transfer of a threshold number (three but not one or two doses) of enriched BALF T_EM_ has a dual effect, decreasing AM numbers and recruiting migratory APC, to the airways and lung. Collectively, the results demonstrate that the host immune system establishes a detour pathway to overcome poor antigen presentation in the lung, by routing bacteria to the spleen for production of antigen. A key step in the detour pathway is the killing of infected AM for the release of bacteria, which can be engulfed and transported by MHC-II^+^ migratory APC. Recruitment of migratory APC to the BALF and lung and their differentiation are therefore central to this pathway.

### 2^0^ T_EM_ show improved heterosubtypic protection against *Mtb*

**Fig. 8.**
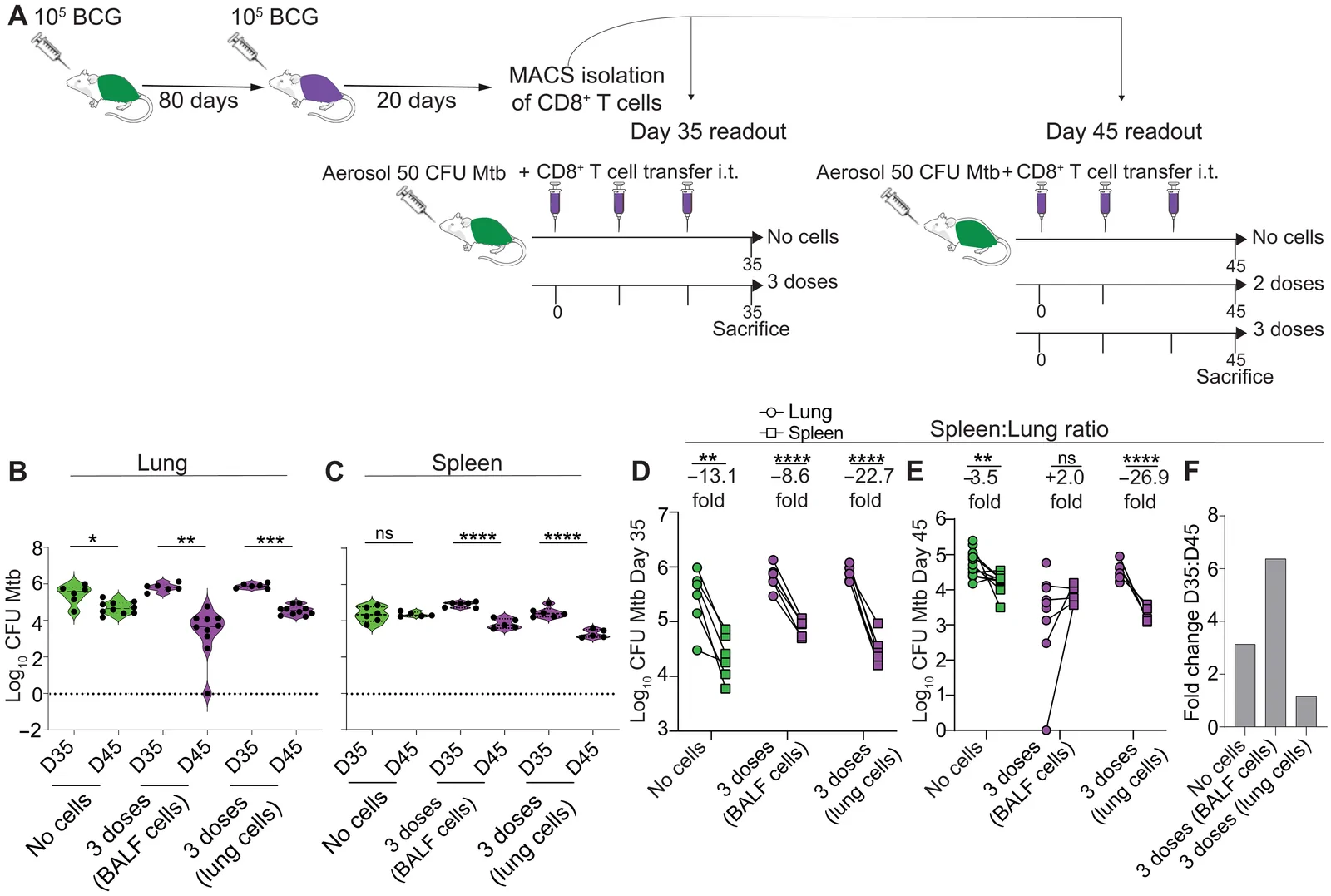
2^0^ T_EM_ show improved heterosubtypic protection against *Mtb*. (**A**) Schematic of experiment. C57BL/6 donor mice were infected i.t. with 10^5^ BCG on day 0 (green). Mice were challenged i.t with 10^5^ BCG 80 days after infection (purple). CD8^+^ T cells were isolated by MACS from BALF and lung 20 days after challenge and transferred i.t. into C57BL/6 recipients (green). Recipients were subsequently infected with 10 to 50 CFU of *Mtb* H37Rv. Recipient mice received either two or three doses of 10^5^ T cells at late time points or three doses 10^5^ T cells at early time points as indicated. Mice were euthanized on day 35 or day 45 after *Mtb* infection. Mice receiving no cells served as controls. (**B** and **C**) Comparison of CFU on day 35 versus day 45 in lung (B) and spleen (C). (**D** and **E**) Fold change in CFU in spleen versus lung on day 35 (D) and day 45 (E). (**F**) Fold change in CFU between day 35 and day 45 in spleen versus lung. Results are presented as individual data points ± SEM (B and C), individual data points (D and E), and fold change (F) from two pooled independent experiments (*n* = 5 to 10 mice per group). Statistical analyses: One-way ANOVA followed by Dunnett’s multiple comparison test (B and C) or Mann-Whitney test (D and E); significant differences are indicated by asterisks: *P < 0.05, **P < 0.01, ***P < 0.001, ****P < 0.0001. Illustration (A) was generated with NIAID NIH BioArt Source images under Public Domain (bioart.niaid.nih.gov).

To address whether adoptive transfer of T_EM_ may also provide protection against TB disease, the above experiment was repeated with adoptive transfer of BALF and lung CD8^+^ T cells from donors as illustrated in Fig. 6A, but this time, recipient mice were challenged with *Mtb* not BCG. BALF and lung CD8^+^ T cells were adoptively transferred in equivalent numbers into naïve recipient mice 12 to 24 hours before *Mtb* aerogenic challenge (Fig. 8A). Some mice received one or two additional doses of CD8^+^ T cells in the late stage. Mice were euthanized on day 35 p.i. (Fig. 8, A to D, and fig. S7, A and B) or 45 p.i. (Fig. 8, A to C and E, and fig. S7, E and F). Mice that did not receive any cells served as control (no cells). *Mtb* clearance was determined by CFU enumeration on agar. Two doses of BALF CD8^+^ T cells and two or three doses of lung CD8^+^ T cells did not reduce CFU (*P* = ns) in the lung on day 35 or 45 p.i. (fig. S7, A and E). Three doses of BALF CD8^+^ T cells significantly reduced CFU by 1.3 log (**P < 0.01) in the lung compared to all other groups on day 45 p.i. (Fig. 8B and fig. S7E), with 80% of the mice showing a bacterial burden that was slightly or significantly reduced compared to the control group (fig. S7, E and I). Overall, *Mtb* spleen CFU was lower than lung CFU on day 35 p.i. regardless of whether BALF or lung CD8^+^ T cells were transferred (Fig. 8, B and C, and fig. S7, A and B). Accordingly, the spleen:lung ratio was <1 (−13.1, −8.6, and −22.7) (Fig. 8D). The spleen:lung ratio for *Mtb* increased 1.5-fold from −13.1- to −8.6-fold on day 35 p.i. when three doses of BALF CD8^+^ T cells were transferred, a phenotype not seen with three doses of lung CD8^+^ T cells or no cells (Fig. 8D). Of note, a similar increase in BCG spleen:lung ratio was detected on day 32 p.i. (from −1.3- to +1.1-fold) when three doses of BALF CD8^+^ T cells were transferred (Fig. 6E). Three doses of BALF CD8^+^ T cells increased bacterial translocation from the lung to the spleen by approximately 3-fold on day 45 p.i., shifting the spleen:lung ratio to a positive value (+2-fold), as compared to zero doses (−3.5-fold) or three doses of lung cells (−26.9-fold) (Fig. 8E). A threefold increase was detected in day 35:day 45 ratio in the BALF T_EM_-treated group (Fig. 8F). All groups of treated mice showed significantly reduced bacterial load ranging from 0.5 to 1.1 log (*P < 0.05, ***P < 0.001, ****P < 0.0001) in the spleen (Fig. 8C and fig. S7F). The number of inflammatory infiltration foci in the lung remained unchanged between control and test groups (*P* = ns) on day 35 p.i., marked by focal granuloma-like lesions (fig. S7, C and D). In contrast, an approximately 40% reduction in the number of inflammatory foci was observed in the treated groups on day 45 p.i., relative to day 35 p.i. (fig. S7D). Furthermore, when the amount of inflammatory infiltration was enumerated across all groups, a significant reduction in infiltration (*P < 0.05) after transfer of three doses of BALF cells was observed on day 45 p.i. relative to day 35 p.i. (fig. S7, G and H). The data show that *Mtb* translocation from the lung to the spleen is accelerated on day 45 p.i. in mice that were adoptively transferred three doses of enriched BALF T_EM_, and this is followed by *Mtb* killing in the spleen (fig. S7F). *Mtb* translocation to the spleen was not evident in the no cell transfer infection timeline (fig. S7, J to M). MPEC isolated 1.5 years after 1^0^ BCG infection and then transferred into recipient mice (fig. S7, N to P) failed to regulate *Mtb* translocation from the lung to the spleen. Together, we concluded that the host immune system uses the same detour pathway to translocate both BCG and *Mtb* to the spleen. Bacterial translocation to the spleen begins after day 20 p.i. following recruitment and differentiation of migratory APC. BALF T_EM_ transfer increases translocation to the spleen on day 32 (BCG) and day 35 (*Mtb*) p.i. *Mtb* killing in the spleen ensues on day 45 p.i. after T_EM_ receive antigenic signaling in the spleen.

## DISCUSSION

An incomplete understanding of T cell–mediated immunity to TB has hindered the development of improved vaccines and immunotherapies for decades. Not only do CD8^+^ memory T cells fail to recognize infected AM early during *Mtb* infection, but long-term persistence of T_EM_ and T_RM_ cells in the respiratory tract is also difficult to achieve, likely to preserve tissue integrity and prevent impairment of gas exchange function. In our study, all three CD8^+^ memory T cell subsets (T_EM_, T_CM_, and T_RM_) increased during the first 20 days of 1^0^ mycobacterial infection; however, this was not accompanied by a reduction in lung bacterial burden. Our data are consistent with studies showing that naïve CD8^+^ T cells undergo massive clonal expansion in a low-antigen environment, resulting in immunodominance and ineffective responses (*28*, *45*). Such immunodominance is not driven by terminal differentiation (*28*), but rather by poor antigen presentation due to phagosomal seclusion by *Mtb*-infected AM (*27*, *41*).

Paradoxically, pulmonary CD8^+^ T_CM_ and T_EM_ numbers were reduced during the 2^0^ response, but pathogen killing was accelerated. This reduction was not a result of attrition but instead due to enhanced migration capability acquired by 2^0^ memory T cells following repeated antigen encounter. Secondary T_EM_ rapidly exited the lung and trafficked to the spleen, where they responded vigorously to antigen. Repeated antigen exposure is known to select for higher-affinity TCRs with improved peptide/MHC binding, resulting in enhanced migration, proliferation, and functional capacity (*46*, *47*). In line with this, 2^0^ T_EM_ in our study mounted robust responses to the immunodominant TB10.4 antigen in the spleen, indicating that recall responses were no longer dwarfed by naïve T cell expansion. While we did not assess TCR repertoires directly, it is plausible that repeated antigen encounter qualitatively reshaped the CD8^+^ memory T cell pool, consistent with reports identifying common TCR specificities enriched in TB controllers versus progressors (*48*).

The effectiveness of CD8^+^ T cell transfer-mediated protection was closely linked to the composition and differentiation state of mononuclear phagocytes in the lung, a feature that is known to change dynamically during *Mtb* infection (*42*, *43*). Our data show that early responses are characterized by abundant undifferentiated monocytes and macrophages, limited migratory DC and moDC populations, poor antigen presentation, nonprotective T_EM_ response, and high bacterial burden, conditions that favor mycobacterial persistence (*42*, *43*). Under these circumstances, adoptive transfer of 2^0^ T_EM_ had little effect. In contrast, as differentiated migratory DC, moDC, and IM numbers accumulated later in infection, bacterial load in the lung decreased and was accompanied by increased bacterial translocation to the spleen. This is consistent with previous work demonstrating that transport of mycobacteria from the lung depends on migratory APC, that *Mtb* grows differently in AM versus IM, and that adoptive transfer of antigen-primed, mature DC at the time of *Mtb* exposure can overcome the delay in T cell recruitment and confer protection (*42*, *49*). Hence, we conclude that transfer of 2^0^ T_EM_ accelerates translocation of bacteria to the spleen, but only once immature monocytes have differentiated into moDC and moDM. 2^0^ T_EM_ on their own are not protective in an environment dominated by immature APC.

**Fig. 9.**
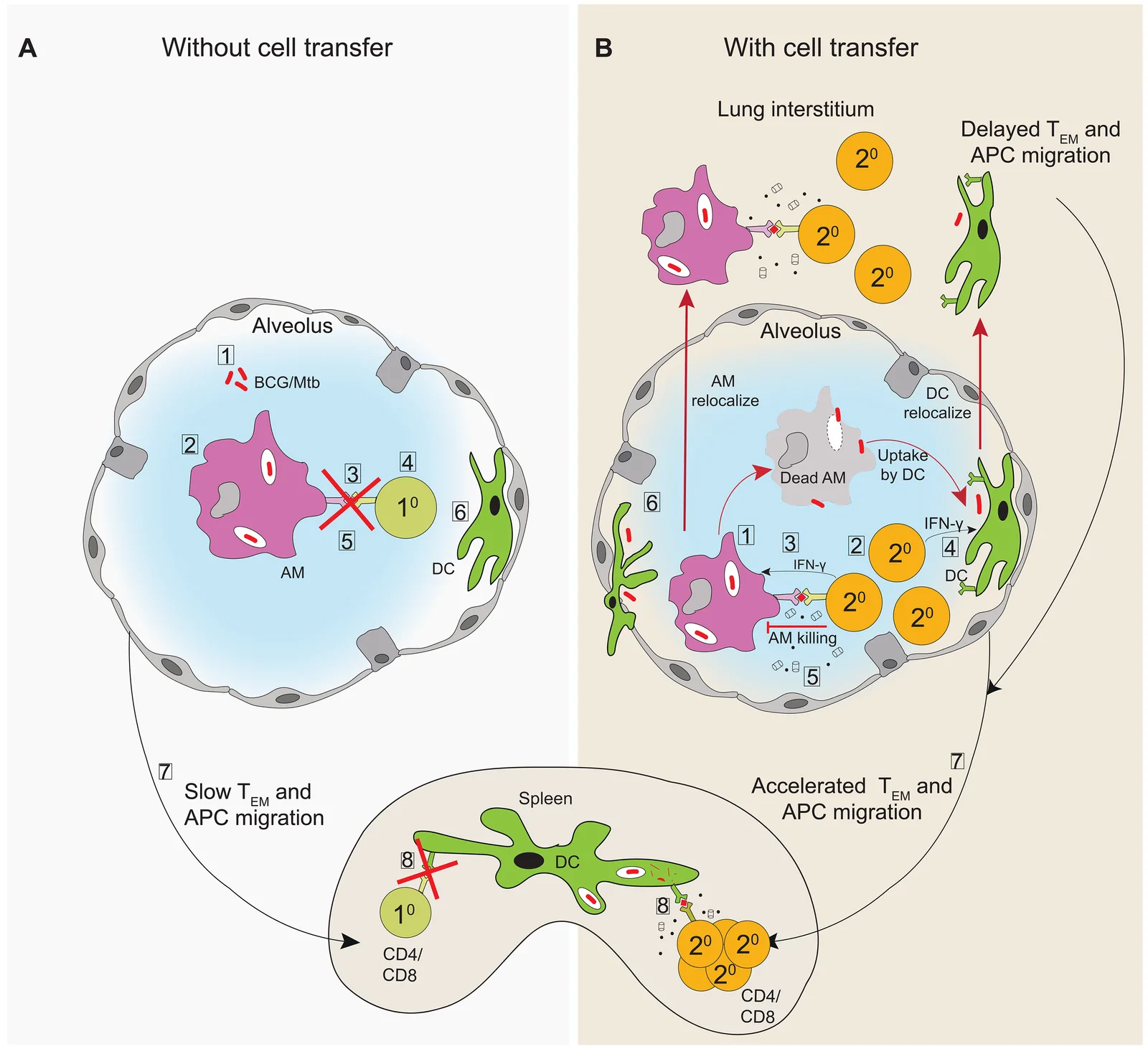
Model - 2^0^ airway T_EM_ promote mycobacterial killing in the spleen. (**A**) (without cell transfer): (1) Transmission of aerosolized bacterial droplets; (2) bacterial containment by AM; (3) limited antigen presentation by AM; (4) 1^0^ T_EM_; (5) limited target recognition and killing of AM; (6) limited migratory APC recruitment; limited bacterial uptake by migratory APC; (7) slow T_EM_ and APC migration; (8) limited antigen processing and presentation by migratory DC; limited target recognition and killing by T_EM_. (**B**) (with cell transfer): (1) Bacterial containment by AM; (2) 2^0^ T_EM_; (3) effective antigen presentation by trained AM; (4) migratory APC recruitment by T_EM_ potentially driven by IFN-γ (needs future experimental validation); (5) target recognition and killing of AM; release of contained bacteria; (6) Bacterial uptake by migratory APC; (7) accelerated migration of T_EM_; accelerated translocation of migratory APC; (8) effective antigen processing and presentation by migratory DC; target recognition and killing by 2^0^ T_EM_ in the spleen. *Mtb* and BCG follow the same cycle. AM must relocalize to the lung interstitium before lung T_EM_ can be activated, resulting in a delay at every step of the cycle. APC, antigen-presenting cell.

Our data reveal a critical “window” (days 20 to 40) of peak T_EM_ migration and show that in the first 20 days of infection, containment of BCG by AM in the lung (*50*) is paralleled by limited antigen presentation, T_EM_ dysfunction, high bacterial load, limited migratory moDC/DC recruitment, and limited BCG translocation to and killing in the spleen. BCG and *Mtb* translocation to the spleen occurs between day 20 and 45 p.i. in no cell control mice. 2^0^ T_EM_ expedite BCG and *Mtb* translocation by at least 20 and 15 days, respectively. By day 60 p.i., T_EM_ and T_CM_ return to the lung, suggesting cyclical migration through the spleen; however, BCG levels are already very low at this stage. Our data suggest that both BCG and *Mtb* may be primarily killed in the spleen. Recycled T_EM_, however, may have an important role in killing *Mtb* in the lung. Of note, the 2^0^ T_EM_ transferred here expressed CXCR3, suggesting that chemokine gradients guided their protective lung infiltration (*51*). Thus, CD8^+^ T_EM_ migration and redistribution appears critical for effective mycobacterial killing. It appears that the host immune system uses a detour pathway to overcome poor antigen presentation in the lung, by routing both BCG and *Mtb* to the spleen for the production of antigen and bacterial killing (Fig. 9).

The history of antigen exposure profoundly shapes the memory CD8^+^ T cell pool, with 2^0^ memory often exhibiting enhanced durability, proliferation, tissue migration, effector function, and hetero-subtypic protection (*32*–*35*). This may explain the cross-protection observed here between BCG-induced T cell memory and *Mtb* challenge. Our data support a model whereby T_EM_ influence *Mtb* mobility, possibly by “coaxing” infected APCs to the spleen for a more conducive environment for antigen presentation and pathogen clearance. Further, adoptive transfer of 2^0^ T_EM_ resulted in 43% killing of AM in the lung, potentially mediated through cytolytic activity or macrophage training via IFN-γ, which has been shown to enhance MHC-II expression and antibacterial immunity in AM (*52*). AM also relocalize from the airway to the lung, which precedes *Mtb* dissemination to other monocyte-derived cells, a process dependent on ESX-1 virulence factors and host IL-1 receptor signaling (*30*). Hence, it is conceivable that the delay in lung T_EM_ migration and function is due to the delayed arrival of antigen via infected AM to the lung interstitium.

Andersen and Urdahl (*11*) have argued that failure of adaptive immunity in TB stems not from a lack of T cell generation, but from delayed, incorrect localization and functionally impaired responses. Activated KLRG1^+^CD4^+^ T cells often become sequestered in nonprotective compartments and fail to access the lung parenchyma where infected macrophages reside (*11*). The KLRG1^+^CD127^low^ CD8^+^ T cells used here are considered short lived (*53*). Thus, how can a short-lived cell provide protection when transferred into a new host? The answer may be simple. A subset of effector-like CD8^+^ T cells expressing KLRG1^hi^CD27^lo^T-bet^hi^ persists into the memory phase and, after boosting, provide superior control of *Listeria monocytogenes* and vaccinia virus, despite limited recall proliferation (*54*). Hence, KLRG1^+^CD8^+^ T cells are not just short lived but, depending on their antigen stimulation history, can retain effector-like properties, persist in the spleen, and become efficient killer cells (*32*).

It is tempting to speculate that the detour pathway via the spleen helps shuttle mycobacteria away from the immunosuppressive environment in the lung. While we did not specifically study T_reg_ activity, 2^0^ T_EM_ may become immune to T_reg_-induced suppression by rapidly leaving the respiratory tract.

Last, our results show that adoptive transfer of airway T_EM_ provides better protection than lung T_EM_. While the reason for this disparity remains unclear, it is conceivable that delayed migration and antigen recognition by lung T_EM_ reflects the time required to relocalize antigen from the airways to the lung interstitium (*30*). Relocalization may also serve to escape the potentially more cytolytic BALF. Positioned at the site of infection, airway T_EM_ retain the capacity to rapidly proliferate and mount recall responses (*55*). Mucosal immunization effectively generates and maintains airway T_EM_ independent of peripheral T cell recruitment (*17*), and their functional plasticity may make them superior to lung parenchyma–derived T_EM_ (*56*, *57*). Collectively, our findings challenge the prevailing paradigm of recall immune responses to mycobacteria and propose an original concept that integrates antigen-experienced T_EM_ migration to the spleen as a critical step to initiate *Mtb* killing. Our results reinforce the idea that BCG should be given mucosally and may help explain why repeated *Mtb* exposure can be protective in natural settings. Hence, strategies aimed at expanding or mobilizing airway T_EM_ through adoptive transfer, as shown here, may overcome current limitations in TB vaccine efficacy. Exploiting these cells for targeted host-directed therapy (*58*, *59*) may not only improve but also expedite early bacterial control in the lung.

## MATERIALS AND METHODS

### Study design

A major barrier to the development of effective vaccine and immunotherapies for TB is the incomplete understanding of TB pathogenesis and the immunological mechanism of protection. Here, we sought to understand how host immune mechanisms eliminate mycobacteria from the respiratory tract. In controlled lab experiments, mice were mucosally infected with *M. bovis* BCG or *Mtb*. Flow cytometry, CFU enumeration and histopathology were used to characterize memory T cell activation and bacterial killing over an extended timeline. Adoptive transfers of airway-derived memory T cells were used to differentiate response of primary and secondary memory T cells and to assess trafficking. Samples were obtained from 5 to 20 animals per group, and experiments were replicated two times.

### Mice

All mice used in this study were on a C57BL/6 background. C57BL/6 female mice aged 6 to 8 weeks were obtained from the Animal Resources Centre (Perth, Australia). *Gt(ROSA)26Sor^tm4(ACTB-tdTomato,-EGFP)Luo^*/J (also known as *mT/mG*) mice were originally from the Jackson Laboratory (*60*) with a substrain donated by S. Mueller (University of Melbourne). This study was carried out in accordance with the guidelines from the National Health and Medical Research Council of Australia. All animal procedures and protocols were approved by the Animal Ethics Committee of James Cook University (A2346 and A2837). All mice were maintained with environmental enrichment under stringent specific pathogen–free conditions in PC2 (BCG vaccination experiments) and PC3 (*Mtb* challenge experiments) animal facilities at the Australian Institute of Tropical Health and Medicine, James Cook University, Australia. Animals were randomly allocated to experimental groups. All animal procedures were performed by trained specialists.

### Bacteria

BCG Danish SSI and *Mtb* H37Rv were cultured in Middlebrook 7H9 broth (BD Biosciences) supplemented with 0.2% glycerol, 0.05% Tween 80, 10% albumin dextrose catalase (ADC) enrichment (BD Biosciences), and relevant antibiotics. Mid-logarithmic [OD_600_ (optical density at 600 nm), 0.6 to 0.8] cultures were harvested, washed in phosphate-buffered saline (PBS), and stored at −80°C until required. Frozen stock vials were thawed, and the bacteria were centrifuged for 12 min at 3500 rpm. The resulting bacterial pellet was resuspended and diluted in PBS to the appropriate density as required for infection (see below).

### Infection

C57BL/6 mice were infected i.t. with 1 × 10^5^ CFU of BCG, as illustrated in schematics. Before intratracheal infections, mice were anesthetized using a 1:16:160 mixture of xylazine, ketamine, and PBS, administered intraperitoneally, or via inhalation of isoflurane. Once anesthetized, mice were suspended by their upper incisors, and 20 μl of bacteria was introduced into the oropharynx. The nostrils were occluded until the inoculum was cleared from the oropharynx. These procedures were conducted on day 0 (1^0^ infection) and day 80 (2^0^ infection) according to the experimental timeline shown in figures. To assess protection against TB, mice were exposed to a low dose of *Mtb* H37Rv (10 to 50 CFU) via aerosol using a GlasCol inhalation exposure system. Determination of the initial aerosol challenge dose involved homogenizing the lungs of five control mice in 1 ml of PBS containing 0.05% Tween 80, 24 hours after infection. The entire homogenized tissue volume was then plated and incubated, and CFU were counted (refer to CFU enumeration below).

### Sample collection

Mice were humanely euthanized either by carbon dioxide asphyxiation or by cervical dislocation. To collect BALF, a small incision was made below the exposed larynx. Using an 18-gauge blunted needle, 1 ml of PBS was flushed into the lungs through the incision and then aspirated. This process was repeated three times to obtain a total of 3 ml of BALF per mouse. Lungs were perfused with PBS via the left ventricle of the heart, and both lungs and spleen were harvested aseptically.

### CFU enumeration

To enumerate BCG colonies, the perfused right lobe of the lung and the spleen were homogenized in 1 ml of PBS containing 0.05% Tween 80, and 100 μl of the homogenate was plated onto Middlebrook 7H11 agar plates enriched with 10% oleic albumin dextrose catalase (OADC) (BD Biosciences). BALF CFU were enumerated by spreading the entire undiluted BALF onto large agar plates with a diameter of 14.5 cm. For *Mtb* CFU enumeration, the perfused right lung was homogenized in 1 ml of PBS with 0.05% Tween 80, and 10-fold serial dilutions (N, 10^−1^, and 10^−2^) were prepared. Aliquots (100 μl) were then plated onto Middlebrook 7H11 agar plates enriched with 10% OADC, ampicillin (20 mg/ml), cycloheximide (50 mg/ml), and 2-thiophenecarboxylic acid hydride (2 mg/ml) (Sigma-Aldrich). The addition of 2-thiophenecarboxylic acid inhibits the growth of BCG while allowing the growth of *Mtb* colonies, enabling the enumeration of *Mtb* colonies exclusively. In all cases, the plates were sealed with parafilm, wrapped in aluminum foil, and aerobically incubated for 4 to 6 weeks at 37°C. Subsequently, the colonies were counted, and the dilution factors were considered when calculating the total bacterial burden of the organs.

### Lung histology

The left lung lobe of mice was perfused, harvested, and fixed in 10% neutral-buffered formalin for 24 hours, followed by storage in 80% ethanol at 4°C. Subsequently, the tissues were processed and embedded in paraffin (HistoCore PEARL, Leica). Sections of 4 μm thickness were cut and transferred onto glass slides (Snowcoat Clipped Corner Slides, Leica). Deparaffinization of the tissues was carried out using xylene and ethanol washes, followed by rehydration, and staining with hematoxylin and eosin using a Leica ST4020 Small Linear Stainer. Images were captured using a dissecting microscope at ×20 magnification. Histopathology and morphometric quantitation were conducted using ImageJ (Fiji). The total lung tissue area and areas of inflammatory changes were determined using the “Freehand selection” tool in ImageJ, enabling manual selection of regions of interest within the images. For quantification of areas with different intensities of cell infiltration (fig. S8), lung areas were identified as either low-intensity infiltration (pink), medium-intensity infiltration (light purple) or high-intensity infiltration (dark purple).

### Flow cytometry

Airway luminal cells were harvested from BALF. Perfused right superior and middle lobes of the lung were used for cell isolation. The method for flow cytometry has been reported (*61*). Mice were euthanized, and their lungs were perfused via the heart with 30 ml of PBS to eliminate blood contaminants from the lung vasculature. The lung tissue was then dissociated into single-cell suspensions using a semiautomated gentleMACS Dissociator following the manufacturer’s instructions (Miltenyi Biotec). These cells were suspended in RPMI 1640 medium supplemented with 10% fetal bovine serum, collagenase VIII, collagenase D (both from Sigma-Aldrich, St. Louis, MO, USA), and DNase I (Sigma-Aldrich), and incubated at 37°C for 30 min. To ensure a homogeneous cell suspension, debris and cell aggregates were removed using MACS SmartStrainers (Miltenyi Biotec). Subsequently, the cells underwent treatment with RBC lysis buffer according to the manufacturer’s instructions (eBioscience). After two washes, the cells were suspended in 2% (v/v) fetal bovine serum in PBS containing anti-CD16/32 (2.4G2) (BD, New Jersey, USA) to block Fc receptors. Fixable Viability Stain 780 (BD Biosciences) was used to exclude dead cells. Samples were then incubated with the H-2Kb *Mtb* TB10.4 tetramer (1:50 dilution) for 1 hour at room temperature and washed with fluorescence-activated cell sorting (FACS) buffer solution. Monomers of H-2Kb *Mtb* TB10.4 4–11 IMYNYPAM were provided by the NIH Tetramer Core Facility, USA. Monomers were tetramerized with Streptavidin-APC (Agilent Technologies) in-house, according to NIH Tetramer Core Facility instructions. The cells were then labeled by incubating with fluorochrome-labeled antibodies (refer to table S1) diluted in FACS buffer for 30 min on ice. Samples were resuspended in 150 μl of blank calibration particles (BD Biosciences) diluted in FACS buffer solution (1:74) and analyzed using a FortessaX20 analyzer (BD Biosciences). Calibration particles ranging from 0.4 to 6.0 μm (BD Biosciences) were used for cell enumeration. FlowJo software (Treestar, Ashland, OR, USA) was used for data analysis.

### T cell isolation and adoptive transfer

T cell isolation was performed as previously described (*61*). Cell isolation was conducted within a biological safety cabinet to ensure sterile conditions during transfer into recipient mice. CD8^+^ T cells were obtained via positive selection by disaggregating lung and BALF cells from BCG-infected C57BL/6 donor mice. These cells were then labeled with CD8-conjugated immunomagnetic beads (Miltenyi Biotec, Bergisch Gladbach, Germany) and sorted using a MACS system (Miltenyi Biotec). Further enrichment was achieved by passing the positive fraction through a second MACS column. Flow cytometry analysis revealed that up to 85% of the cells in the positive fraction were CD3^+^CD8^+^ T cells, while approximately 5% of CD8^+^ T cells were found in the CD8^−^ fraction. For adoptive transfer experiments, C57BL/6 recipient mice aged 6 to 8 weeks were initially infected i.t. with 10^5^ CFU of BCG. Subsequently, 10^5^ MACS-purified CD8^+^ T cells were transferred i.t. into recipient mice. Additional cell transfer doses were administered i.t. as depicted in the figures. Mice that did not receive cells served as the negative control. For mT/mG cell transfer, airway luminal cells and lung cells were processed as described above for flow cytometry. Equivalent numbers of total unfractionated airway luminal and lung cells were transferred into C57BL/6 recipient mice.

### Statistical analyses

Correlations were calculated and plotted using Prism (GraphPad Prism v10, San Diego, CA, USA). For comparison of two groups, the Student’s *t* test (normally distributed) or the Mann-Whitney *U* test (not normally distributed) was used. For multiple comparisons, one-way analysis of variance (ANOVA) was used. A difference between groups was considered significant if *P* < 0.05.

## Supplementary Materials

### This PDF file includes:

Figs. S1 to S8 Table S1 References
