# Supplementary material for: Antigen-experienced airway CD8+ effector memory T cells promote a detour pathway for mycobacterial killing in the spleen
Source: Sci Adv. 2026 Aug 21;12(34):eaec7792. doi: 10.1126/sciadv.aec7792 (PMC13496176; doi:10.1126/sciadv.aec7792)
Supplement: Supplementary file 1 [file sciadv.aec7792_sm.pdf]

Science Advances
AAAS

# Supplementary Materials for

## Antigen-experienced airway CD8$^+$ effector memory T cells promote a detour pathway for mycobacterial killing in the spleen

Julia Seifert *et al.*

Corresponding author: Saparna Pai, saparna.pai@gmail.com; Andreas Kupz, andreas.kupz@jcu.edu.au

*Sci. Adv.* **12**, eaec7792 (2026)
DOI: 10.1126/sciadv.aec7792

**This PDF file includes:**

Figs. S1 to S8
Table S1
References

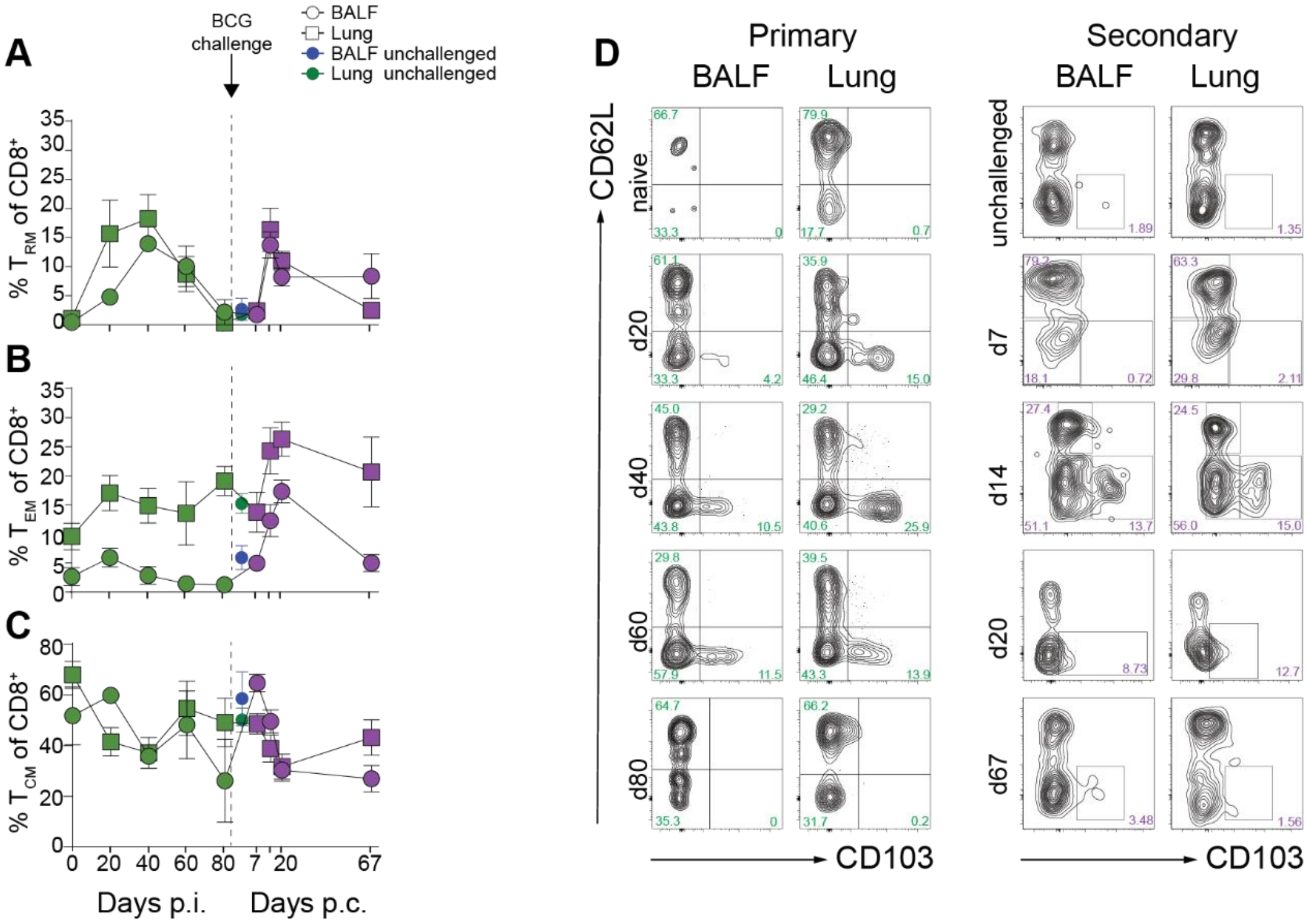


**Figure S1: Percentages of pulmonary CD8$^{+}$ memory T cells over time. (A-C)** C57BL/6 mice were infected i.t. with $10^5$ BCG ($1^0$ infection in green) or left uninfected (day 0 or naïve). Some mice were challenged i.t. with $10^5$ BCG 80 days after infection ($2^0$ infection in purple) or left unchallenged. Proportion of $T_{RM}$ (**A**), $T_{EM}$ (**B**) and $T_{CM}$ (**C**) within CD8$^{+}$ T cells, enumerated at various time-points in the BALF (circle) and lung parenchyma (square) using flow cytometric analysis. (**D**) Representative flow plots for the enumeration of CD62L$^{-}$CD103$^{+}$ $T_{RM}$ cells. Results are presented as data means ± SEM (**A-C**) and representative flow plots (**D**) from at least 2 pooled independent experiments (n = 3-16 mice per group). p.i., post infection; p.c., post challenge. Supplementary to Figure 1.

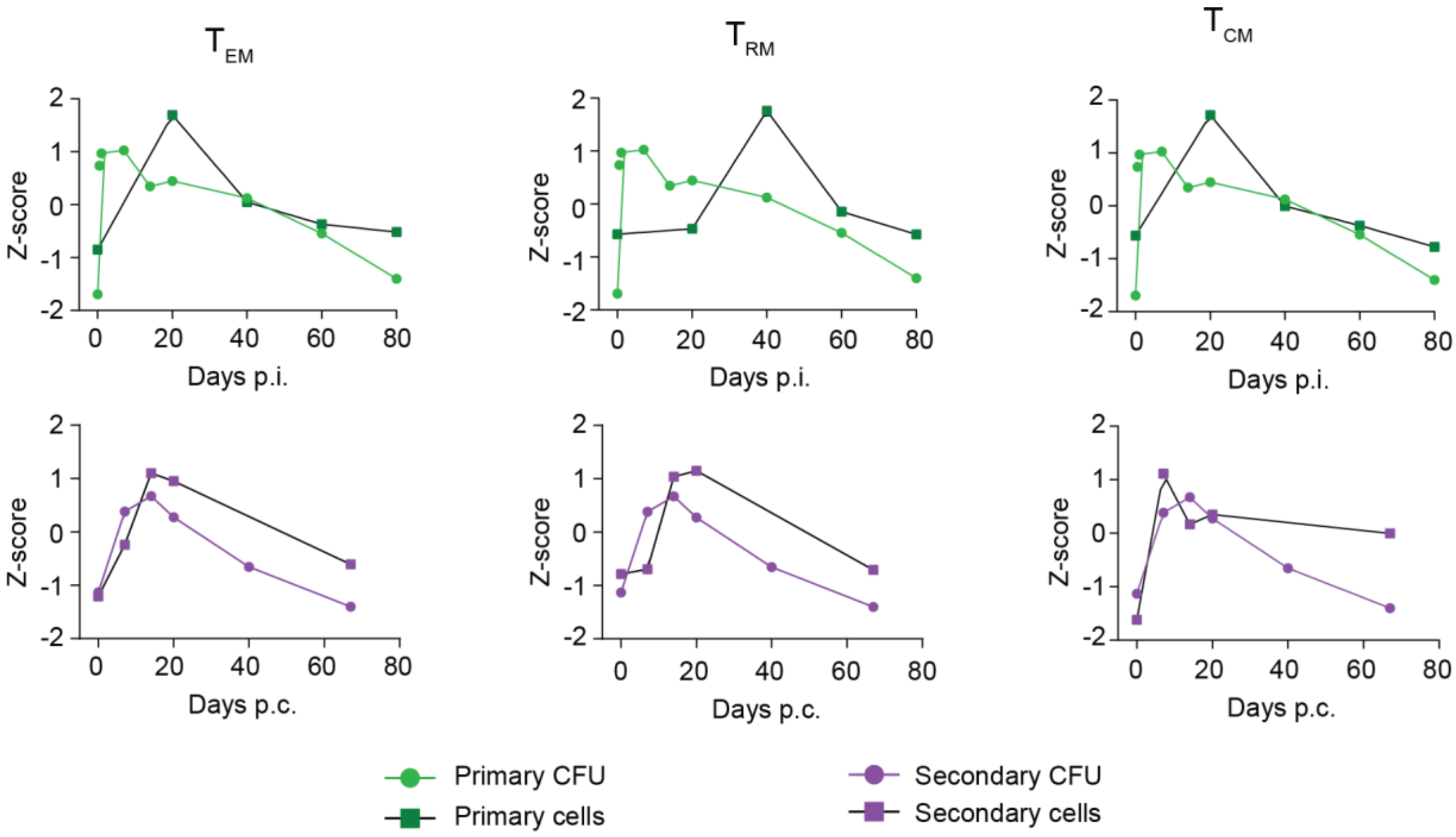


**Figure S2: Z-score-based correlations of CFU and cell numbers over time.** C57BL/6 mice were infected i.t. with $10^5$ BCG and re-challenged i.t. with $10^5$ BCG 80 days after infection. Best fit spline curves showing z-score CFU (circle), as well as $T_{RM}$, $T_{EM}$ and $T_{CM}$ numbers (squares) in lung over time during $1^0$ (green) and $2^0$ (purple) infection. Results are presented as data means from at least 2 pooled independent experiments (n = 3-16 mice per group). p.i., post infection; p.c., post challenge. Supplementary to Figure 2.

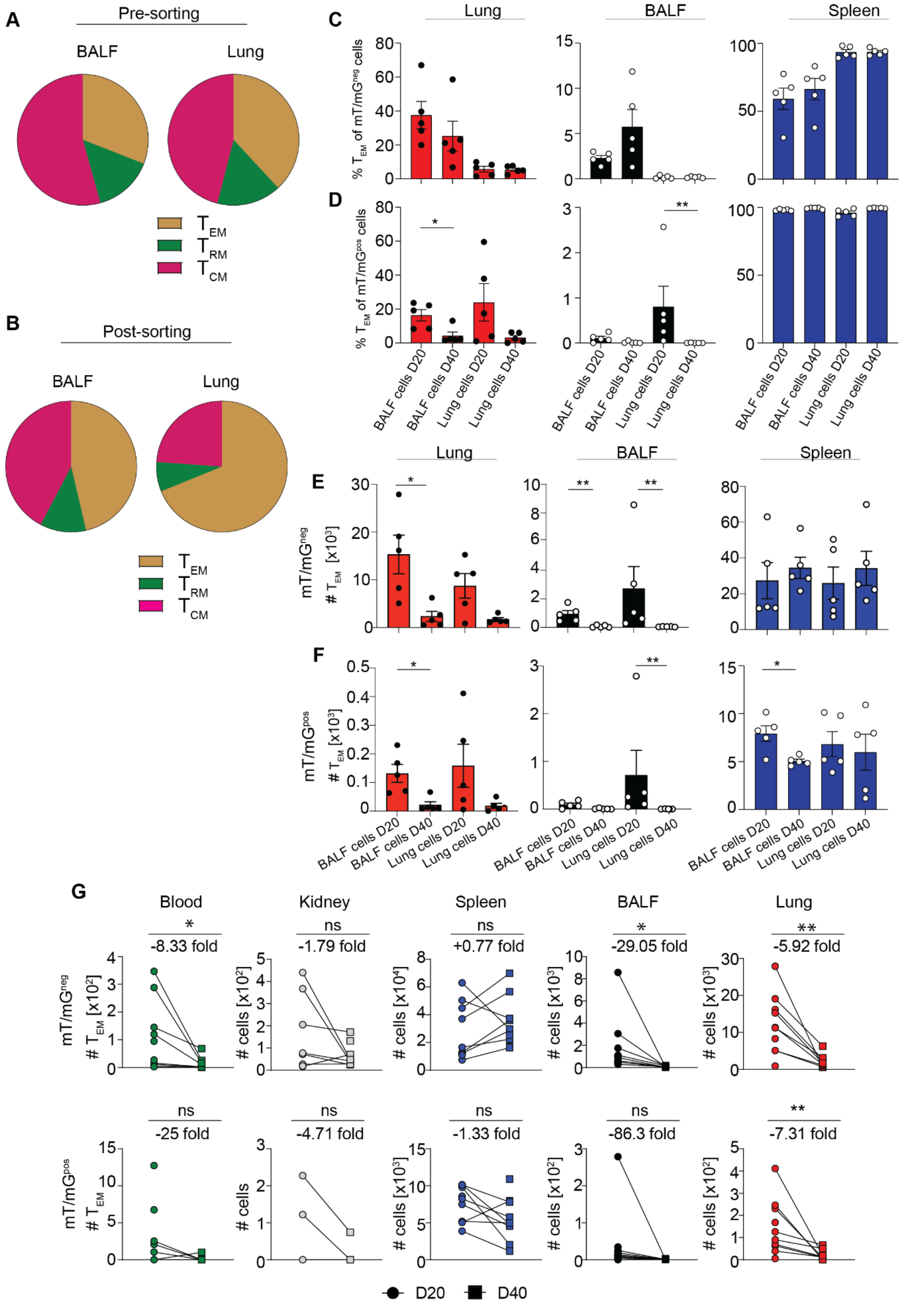


**Figure S3: Migration dynamics of CD8$^{+}$ memory T cells.** (**A-B**) C57BL/6 mice were

infected as shown in Fig. 6A schematic. Proportion of $T_{RM}$, $T_{EM}$ and $T_{CM}$ in BALF and lung donor cells at 20 days p.c. before (**A**) and after (**B**) MACS sorting. (**C-F**) mT/mG mice were infected as shown in Fig. 5A schematic. Percentage (**C, D**) and absolute number (**E, F**) of mT/mG$^{pos}$ $T_{EM}$ and mT/mg$^{neg}$ $T_{EM}$ in lung, BALF and spleen at 20 and 40 days after cell transfer. (**G**) Fold change of mT/mG$^{pos}$ $T_{EM}$ and mT/mG$^{neg}$ $T_{EM}$ between day 20 and day 40 after cell transfer. Results are presented as individual data points ± SEM (**C-F**), individual data points (**G**) and pie charts **(A-B)** from 2 pooled independent experiments (n = 5 mice per group). Statistical analyses: One-way ANOVA followed by Dunnet's multiple comparison test or Mann-Whitney test; significant differences are indicated by asterisks: * $p < 0.05$, ** $p < 0.01$, *** $p < 0.001$, **** $p < 0.0001$. Supplementary to Figure 4.

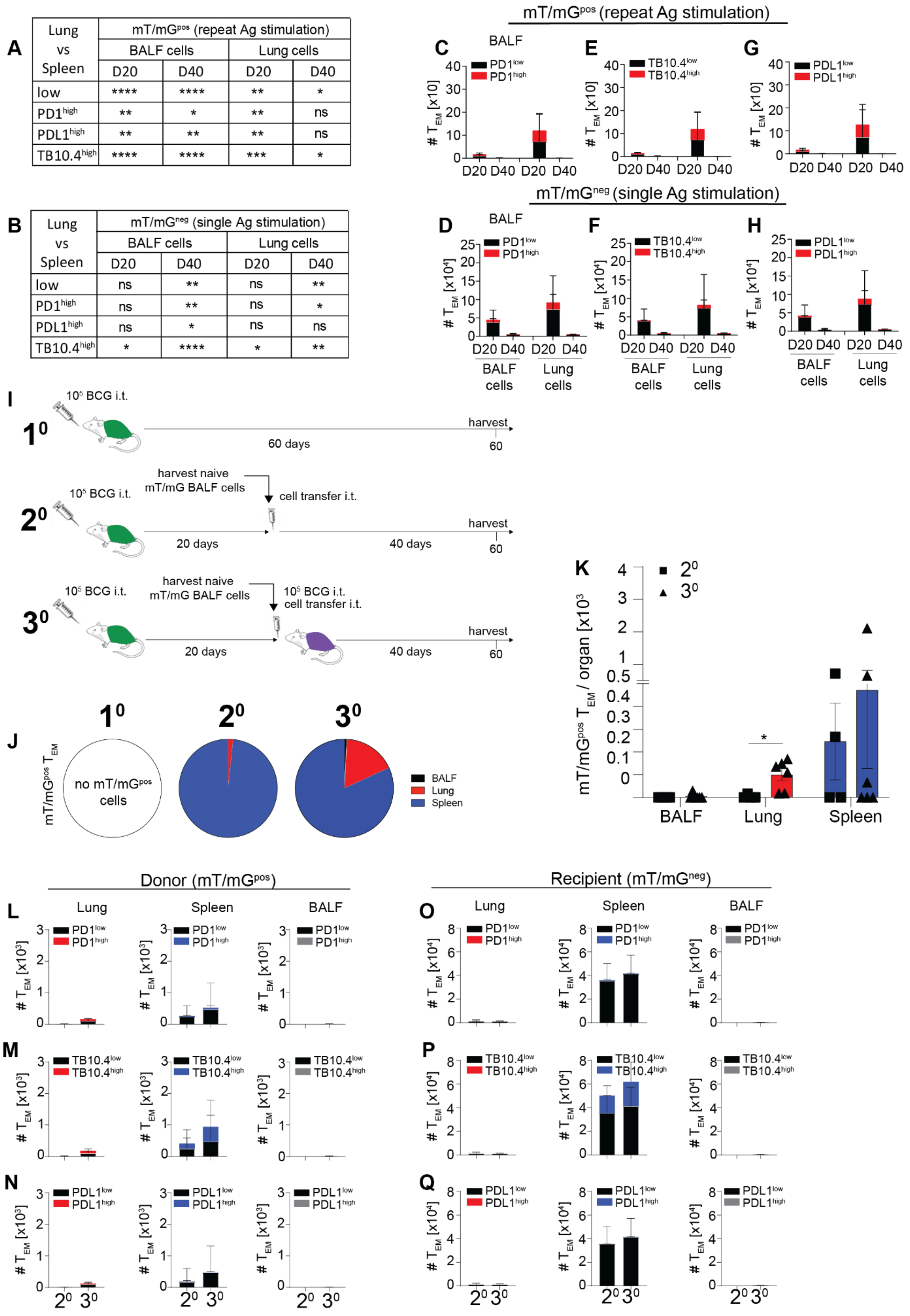


**Figure S4: Marker expression of donor and recipient cells. (A, B)** Statistical comparison

between lung and spleen of mT/mG$^{pos}$ cells after repeat antigen stimulation **(A)** and mT/mG$^{neg}$ cells after single antigen stimulation **(B)**. (**C, D**) Number of PD1$^{hi}$ (red) and PD1$^{lo}$ (black) mT/mG$^{pos}$ cells after repeat antigen stimulation (**C**) and mT/mG$^{neg}$ cells after single antigen stimulation (**D**) in BALF. (**E, F)** Number of TB10.4$^{hi}$ (red) and TB10.4$^{lo}$ (black) mT/mG$^{pos}$ cells after repeat antigen stimulation (**E**) and mT/mG$^{neg}$ cells after single antigen stimulation (**F**) in BALF. (**G, H**) Number of PDL1$^{hi}$ (red) and PDL1$^{lo}$ (black) mT/mG$^{pos}$ cells after repeat antigen stimulation (**G**) and mT/mG$^{neg}$ cells after single antigen restimulation (**H**) in BALF. (**I**) Schematic of experiment. C57BL/6 donor mice were infected i.t. with $10^5$ BCG on day 0. Mice were adoptively transferred with cells harvested from mT/mG donor mice 20 days p.i. Mice were either challenged with BCG ($3^0$ BCG+mT/mG BALF+BCG) or left unchallenged ($2^0$ BCG+mT/mG BALF). Control mice received no cells ($1^0$ BCG). All groups of mice were sacrificed 60 days p.i. Total BALF cells, lung cells and splenocytes were collected, and analyzed by flow cytometry. (**J**) Proportion of mTmG$^{pos}$ $T_{EM}$ in BALF, lung and spleen. **(K)** Number of mT/mG$^{pos}$ cells at time of organ harvest. **(L-Q)** Number of PD1 (**L, O**), PDL1 (**N, Q**) and TB10.4 (**M, P**) high and low cells in lung, spleen and BALF of donors and recipients. Results are presented as statistical comparisons (**A, B**), data means ± SEM (**C-H, L-Q**), individual data points ± SEM (**K**) and pie charts (**J**) from 2 pooled independent experiments (n = 4-6 mice per group). Statistical analyses: One-way ANOVA followed by Dunnet's multiple comparison test (**A-B**) or Mann-Whitney test (**K**); significant differences are indicated by asterisks: * $p < 0.05$, ** $p < 0.01$, *** $p < 0.001$, **** $p < 0.0001$. Illustration (**I**) generated with NIAID NIH BioArt Source images (bioart.niaid.nih.gov). Supplementary to Figure 5.

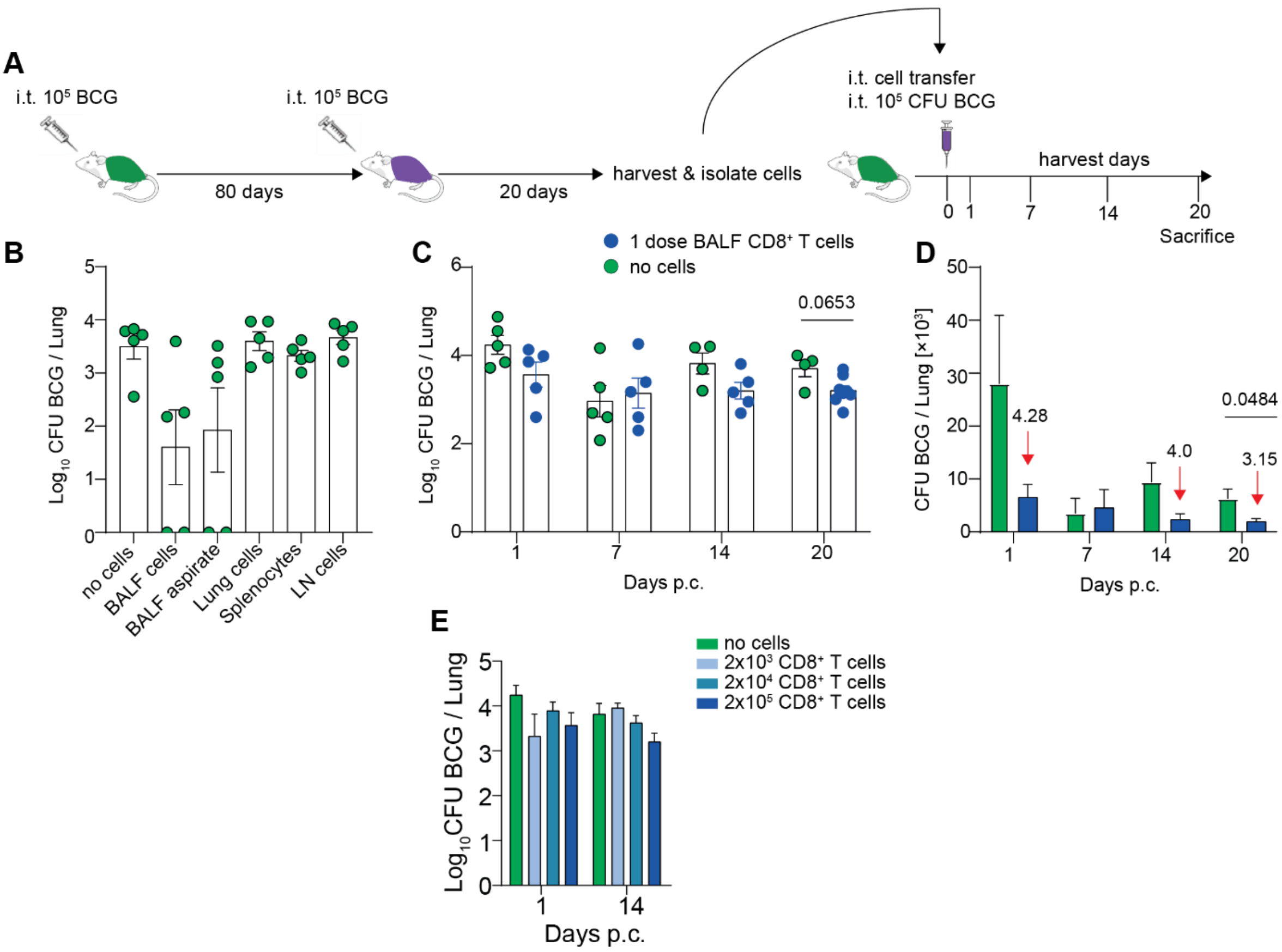


**Figure S5: BCG bacterial burden following adoptive cell transfer.** **(A)** Schematic of experiment. C57BL/6 donor mice were infected i.t. with $10^5$ BCG on day 0. Mice were challenged i.t. with $10^5$ BCG 80 days after infection. Total BALF cells, BALF aspirate, total lung cells, total splenocytes or total LN cells were collected (**B**) or CD8$^+$ T cells were isolated by MACS from BALF and lung (**C, D**), 20 days after challenge and transferred i.t. into C57BL/6 recipients that were subsequently infected i.t. with $10^5$ BCG. (**B**) BCG CFU in lung at 20 days after total cell transfer. (**C, D**) Log transformed (**C**) and linear (**D**) BCG CFU in the lung at 1, 7, 14 and 20 days after transfer of MACS-sorted CD8$^+$ T cells. Fold change values shown with red arrows. **(E)** Log transformed BCG CFU in the lung at 1 and 14 days after transfer of different amounts of MACS-sorted CD8$^+$ T cells. Results are presented as individual data points ± SEM (**B, C**) and data means ± SEM (**D, E**) from one experiment (n = 5-8 mice per group). Statistical analyses: One-way ANOVA followed by Dunnet's multiple comparison test; statistical differences are indicated by *p* value. Illustration (**A**) generated with NIAID NIH BioArt Source images (bioart.niaid.nih.gov). Supplementary to Figure 6.

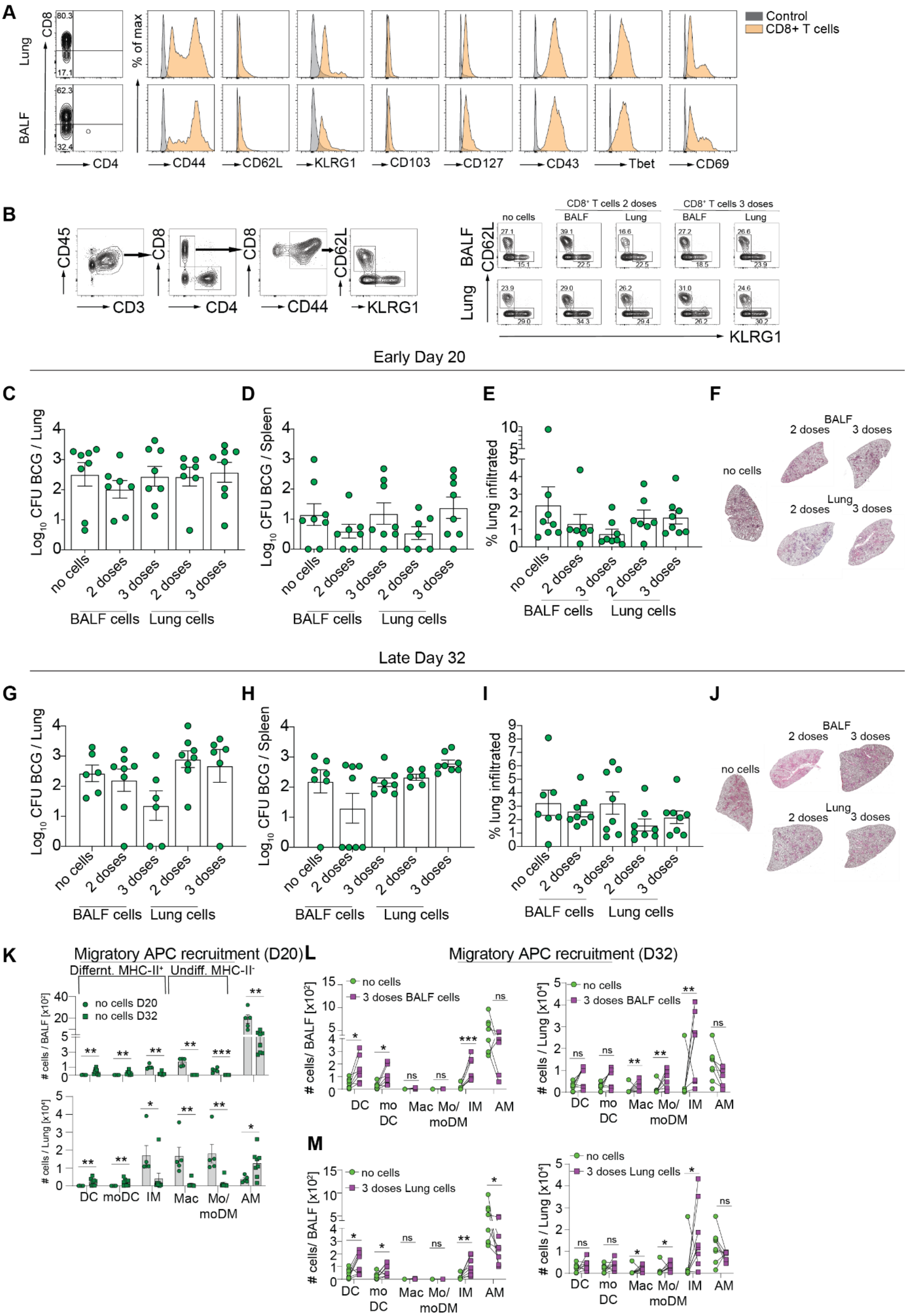
A
Lung
BALF
CD8
CD4
% of max
CD44
CD62L
KLRG1
CD103
CD127
CD43
Tbet
CD69
Control
CD8+ T cells
B
CD45
CD3
CD8
CD4
CD44
CD62L
KLRG1
no cells
CD8⁺ T cells 2 doses
CD8⁺ T cells 3 doses
BALF
Lung
Early Day 20
C
D
E
F
Log₁₀ CFU BCG / Lung
Log₁₀ CFU BCG / Spleen
% lung infiltrated
no cells
2 doses
3 doses
BALF cells
Lung cells
Late Day 32
G
H
I
J
K
Migratory APC recruitment (D20)
Differnt. MHC-II⁺
Undiff. MHC-II⁻
no cells D20
no cells D32
# cells / BALF [x10²]
# cells / Lung [x10⁴]
DC
moDC
IM
Mac
Mo/ moDM
AM
L
Migratory APC recruitment (D32)
no cells
3 doses BALF cells
M
3 doses Lung cells
ns

**Figure S6: Phenotypic data, bacterial burden, lung histopathology and migratory APC numbers in BCG infection model. (A)** Mice were infected as shown in **Fig. 6A** schematic. Surface marker expression profile of $CD8^+$ T cells isolated from lung and BALF by MACS purification. (**B**) Gating strategy used to identify $T_{CM}$ and $T_{EM}$ in the BALF and lung of recipients with and without cell transfer. (**C, D**) BCG CFU in lung (**C**) and spleen (**D**) after early cell transfer. (**G, H**) BCG CFU in lung (**G**) and spleen (**H**) after late cell transfer. (**E, I**) Percent of dense lung infiltration afer early cell transfer (**E**) or late cell transfer (**I**). Representative H&E images for early (**F**) and late (**J**) cell transfer. **(K)** Comparison of DC, moDC, Mac, moDM, IM and AM numbers in BALF and lung at day 20 and day 32 following BCG infection without cell transfer. **(L, M)** Comparison of DC, moDC, Mac, moDM, IM and AM numbers in BALF and lung following transfer of 3 doses of BALF cells **(L)** or lung cells **(M)** vs no cell transfer at day 32. Results are presented as individual data points ± SEM (**C-E, G-I, K, L, M**), representative images (**F, J**), representative histogram (**A**) and representative flow plots **(B)** from 2 pooled independent experiments (n = 3-8 mice per group). Statistical analyses: One-way ANOVA followed by Dunnet's multiple comparison test or Mann-Whitney test. Significant differences are indicated by asterisks: * $p < 0.05$, ** $p < 0.01$, *** $p < 0.001$. Supplementary to Figure 6.

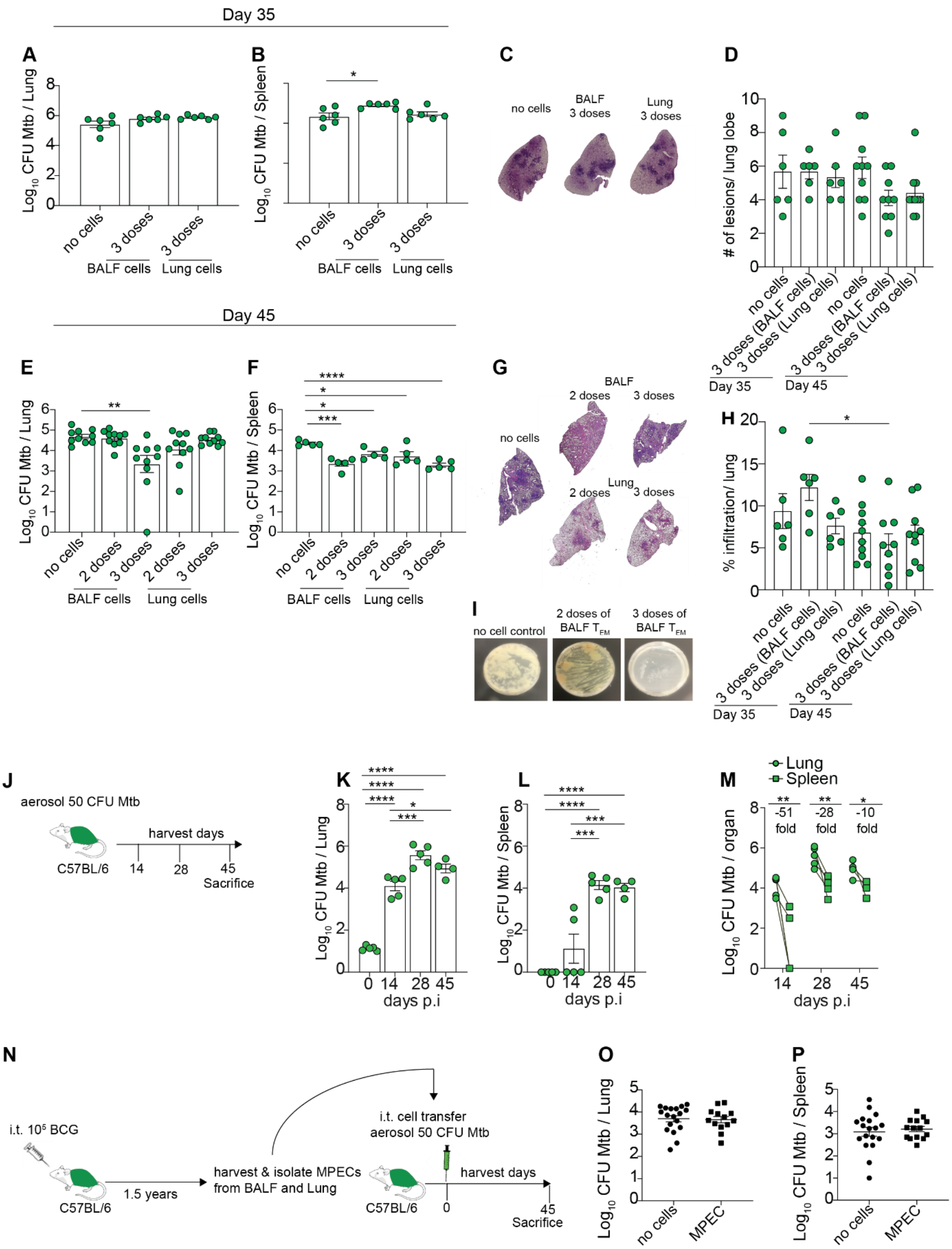


**Figure S7: Bacterial burden and lung histopathology in *Mtb* infection model. (A, B)** *Mtb* CFU in lung (**A**) and spleen (**B**) at day 35 after cell transfer. (**E, F**) *Mtb* CFU in lung (**E**) and spleen (**F**) at day 45 after cell transfer. (**C, G**) Representative H&E images for day 35 (**C**) and

day 45 (**G**) cell transfer. (**D, H**) Number of lesions per lung lobe (**D**) and percent of dense lung infiltration (**H**) at day 35 or day 45 after *Mtb* infection (**I**) Representative images of agar plates from recipient groups. (**J**) Schematic of *Mtb* infection. CFU numbers (**K, L**) and fold change in CFU in lung (**K**), spleen (**L**) and spleen vs lung (**M**) at 14, 28 and 45 days after aerosol *Mtb* infection. (**N**) C57BL/6 donor mice were infected i.t. with BCG on day 0. $CD8^+$ memory precursor effector cells (MPECs) were isolated by MACS from BALF and lung 1.5 years after BCG infection and transferred i.t. into C57BL/6 recipients that were subsequently infected with 10-50 cfu of *Mtb* H37Rv. (**O, P**) CFU in lung (**O**) and spleen (**P**) at 45 days after *Mtb* infection. Results are presented as individual data points ± SEM (**A-B, E-F, D, H, K, L, O, P**), individual data points (**M**), representative images (**C, G**), and photos of representative agar plates (**I**) from at least 2 pooled independent experiments (n = 5-19 mice per group). Statistical analyses: One-way ANOVA followed by Dunnet's multiple comparison test; significant differences are indicated by asterisks: * $p < 0.05$, ** $p < 0.01$, *** $p < 0.001$, **** $p < 0.0001$. Data shown in (**J-M**) reproduced from Sathkumara *et al*. 2020, PNAS, Fig S4E (*62*). Illustration (**J, N**) generated with NIAID NIH BioArt Source images (bioart.niaid.nih.gov). Supplementary to Figure 8.

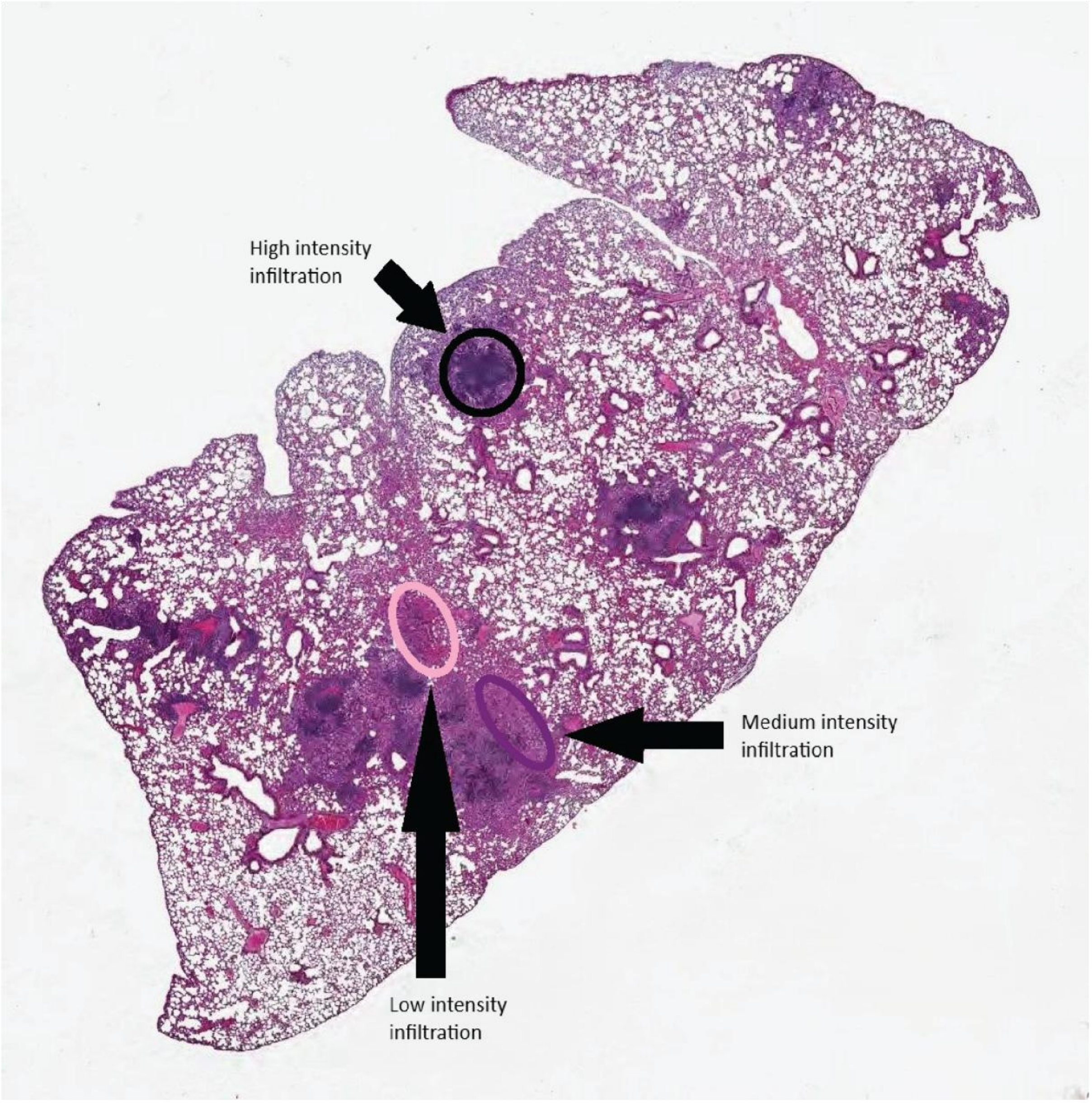


**Figure S8: Different grades of inflammatory infiltrate.** Representative H&E image of a left lung lobe with areas of low intensity, medium intensity and high intensity infiltration highlighted in pink, purple and black, respectively.

**Supplementary Table 1:** List of antibodies used for flow cytometry.

| Antigen | Fluorochrome | Clone |
|---|---|---|
| CD43 | BV788 | S7 |
| CD3 | PE-CF594 | 145-2C11 |
| CD45.2 | BV650 | 104 |
| CD44 | BV421 | IM7 |
| CD8 | BV510 | 53.6-7 |
| CD4 | BUV395 | GK1.5 |
| CD127 | PerCP-Cy5.5 | A7R34 |
| CD62L | PE-Cy7 | MEL-14 |
| CD69 | PE-CF594 | H1.2F3 |
| CD3 | AF700 | 500A2 |
| CD103 | APC | M290 |
| PD-1 | PE | J43 |
| PDL-1 | AF660 | MIH5 |
| TB10.4 | FITC | H-2Kb |
| CD27 | APC | LG.7F9 |
| CD183 | CXCR3 BV786 | CXCR3-173 |
| CD11c | PeCy7 | HL3 |
| SiglecF | PE | E50-2440 |
| CD206 | AF647 | MR5D3 |
| CD11b | BV421 | M1/70 |
| KLRG1 | AF488 | 2F1 |
| F4/80 | BV650 | T45-2342 |
| IA-IE (MHC-II) | BV711 | M5/114.15.2 |
| Ly6C | FITC | AL-21 |
| T-bet | efluor660 | eBio4B10(4B10) |
| CD11a | PerCP-Cy5.5 | H155-78 |
| Ly6G | BUV395 | 1A8 |

**REFERENCES**


1. World Health Organization, Global Tuberculosis Report 2024 (2025); http://who.int/tb/publications/global_report/en/.

2. P. Mangtani, I. Abubakar, C. Ariti, R. Beynon, L. Pimpin, P. E. Fine, L. C. Rodrigues, P. G. Smith, M. Lipman, P. F. Whiting, J. A. Sterne, Protection by BCG vaccine against tuberculosis: A systematic review of randomized controlled trials. *Clin. Infect. Dis.* **58**, 470–480 (2014).

3. B. P. Ndiaye, F. Thienemann, M. Ota, B. S. Landry, M. Camara, S. Dieye, T. N. Dieye, H. Esmail, R. Goliath, K. Huygen, V. January, I. Ndiaye, T. Oni, M. Raine, M. Romano, I. Satti, S. Sutton, A. Thiam, K. A. Wilkinson, S. Mboup, R. J. Wilkinson, H. McShane, Safety, immunogenicity, and efficacy of the candidate tuberculosis vaccine MVA85A in healthy adults infected with HIV-1: A randomised, placebo-controlled, phase 2 trial. *Lancet Respir. Med.* **3**, 190–200 (2015).

4. M. D. Tameris, M. Hatherill, B. S. Landry, T. J. Scriba, M. A. Snowden, S. Lockhart, J. E. Shea, J. B. McClain, G. D. Hussey, W. A. Hanekom, H. Mahomed, H. McShane, Safety and efficacy of MVA85A, a new tuberculosis vaccine, in infants previously vaccinated with BCG: A randomised, placebo-controlled phase 2b trial. *Lancet* **381**, 1021–1028 (2013).

5. K. B. Urdahl, S. Shafiani, J. D. Ernst, Initiation and regulation of T-cell responses in tuberculosis. *Mucosal Immunol.* **4**, 288–293 (2011).

6. M. M. Tonaco, J. D. Moreira, F. F. C. Nunes, C. M. G. Loures, L. R. Souza, J. M. Martins, H. R. Silva, A. H. R. Porto, V. Toledo, S. S. Miranda, T. Guimaraes, Evaluation of profile and functionality of memory T cells in pulmonary tuberculosis. *Immunol. Lett.* **192**, 52–60 (2017).

7. C. Perdomo, U. Zedler, A. A. Kühl, L. Lozza, P. Saikali, L. E. Sander, A. Vogelzang, S. H. E. Kaufmann, A. Kupz, Mucosal BCG vaccination induces protective lung-resident memory T cell populations against tuberculosis. *MBio* **7**, e01686-16 (2016).

8. S. M. Kaech, J. T. Tan, E. J. Wherry, B. T. Konieczny, C. D. Surh, R. Ahmed, Selective expression of the interleukin 7 receptor identifies effector CD8 T cells that give rise to long-lived memory cells. *Nat. Immunol.* **4**, 1191–1198 (2003).

9. M. F. Bachmann, P. Wolint, K. Schwarz, P. Jäger, A. Oxenius, Functional properties and lineage relationship of $CD8^+$ T cell subsets identified by expression of IL-7 receptor α and CD62L. *J. Immunol.* **175**, 4686–4696 (2005).

10. E. Lefrançais, D. Hudrisier, O. Neyrolles, S. M. Behar, J. D. Ernst. Finding and filling the knowledge gaps in mechanisms of T cell-mediated TB immunity to inform vaccine design. *Nat. Rev. Immunol.* ***25***, 798–815 (2025).

11. P. Andersen, K. B. Urdahl, TB vaccines; promoting rapid and durable protection in the lung. *Curr. Opin. Immunol.* **35**, 55–62 (2015).

12. K. M. Huster, C. Stemberger, D. H. Busch, Protective immunity towards intracellular pathogens. *Curr. Opin. Immunol.* **18**, 458–464 (2006).

13. A. J. Wolf, L. Desvignes, B. Linas, N. Banaiee, T. Tamura, K. Takatsu, J. D. Ernst, Initiation of the adaptive immune response to *Mycobacterium tuberculosis* depends on antigen production in the local lymph node, not the lungs. *J. Exp. Med.* **205**, 105–115 (2008).

14. A. J. Wolf, B. Linas, G. J. Trevejo-Nuñez, E. Kincaid, T. Tamura, K. Takatsu, J. D. Ernst, *Mycobacterium tuberculosis* infects dendritic cells with high frequency and impairs their function in vivo. *J. Immunol.* **179**, 2509–2519 (2007).

15. J. D. Ernst, The immunological life cycle of tuberculosis. *Nat. Rev. Immunol.* **12**, 581–591 (2012).

16. R. Zander, D. Schauder, G. Xin, C. Nguyen, X. Wu, A. Zajac, W. Cui, $CD4^+$ T cell help is required for the formation of a cytolytic $CD8^+$ T cell subset that protects against chronic infection and cancer. *Immunity* **51**, 1028–1042.e4 (2019).

17. M. Jeyanathan, J. Mu, S. McCormick, D. Damjanovic, C. L. Small, C. R. Shaler, K. Kugathasan, Z. Xing, Murine airway luminal antituberculosis memory CD8 T cells by mucosal immunization are maintained via antigen-driven in situ proliferation, independent of peripheral T cell recruitment. *Am. J. Respir. Crit. Care Med.* **181**, 862–872 (2010).

18. D. L. Woodland, I. Scott, T cell memory in the lung airways. *Proc. Am. Thorac. Soc.* **2**, 126–131 (2005).

19. A. C. Schmidt, L. Fairlie, E. Hellström, A. L. K. Kany, K. Middelkoop, K. Naidoo, G. Nair, A. Gela, E. Nemes, T. J. Scriba, A. Cinar, N. Frahm, R. Mogg, D. Kaufman, M. W. Dunne, M. Hatherill, BCG revaccination for the prevention of *Mycobacterium tuberculosis* infection. *N. Engl. J. Med.* **392**, 1789–1800 (2025).

20. K. Dijkman, C. C. Sombroek, R. A. W. Vervenne, S. O. Hofman, C. Boot, E. J. Remarque, C. H. M. Kocken, T. H. M. Ottenhoff, I. Kondova, M. A. Khayum, K. G. Haanstra, M. P. M. Vierboom, F. A. W. Verreck, Prevention of tuberculosis infection and disease by local BCG in repeatedly exposed rhesus macaques. *Nat. Med.* **25**, 255–262 (2019).

21. A. M. Cadena, F. F. Hopkins, P. Maiello, A. F. Carey, E. A. Wong, C. J. Martin, H. P. Gideon, R. M. DiFazio, P. Andersen, P. L. Lin, S. M. Fortune, J. L. Flynn, Concurrent infection with *Mycobacterium tuberculosis* confers robust protection against secondary infection in macaques. *PLOS Pathog.* **14**, e1007305 (2018).

22. C. A. Janeway, P. Travers, M. Walport, M. J. Shlomchik, “Chapter 10: Immunological memory,” in *Immunobiology: The Immune System in Health and Disease* (Garland Science, ed. 5, 2001).

23. J. M. Schenkel, D. Masopust, Tissue-resident memory T cells. *Immunity* **41**, 886–897 (2014).

24. F. Sallusto, J. Geginat, A. Lanzavecchia, Central memory and effector memory T cell subsets: Function, generation, and maintenance. *Annu. Rev. Immunol.* **22**, 745–763 (2004).

25. M. Zanetti, S. P. Schoenberger, Eds. *Memory T Cells*, vol. 684 of *Advances in Experimental Medicine and Biology* (Springer Science+Business Media, 2010).

26. E. R. Mehlhop-Williams, M. J. Bevan, Memory CD8$^+$ T cells exhibit increased antigen threshold requirements for recall proliferation. *J. Exp. Med.* **211**, 345–356 (2014).

27. U. E. Schaible, F. Winau, P. A. Sieling, K. Fischer, H. L. Collins, K. Hagens, R. L. Modlin, V. Brinkmann, S. H. Kaufmann, Apoptosis facilitates antigen presentation to T lymphocytes through MHC-I and CD1 in tuberculosis. *Nat. Med.* **9**, 1039–1046 (2003).

28. S. M. Carpenter, C. Nunes-Alves, M. G. Booty, S. S. Way, S. M. Behar, A higher activation threshold of memory CD8$^+$ T cells has a fitness cost that is modified by TCR affinity during tuberculosis. *PLOS Pathog.* **12**, e1005380 (2016).

29. S. C. Wassmer, T. F. de Koning-Ward, G. E. R. Grau, S. Pai, Unravelling mysteries at the perivascular space: A new rationale for cerebral malaria pathogenesis. *Trends Parasitol.* **40**, 28–44 (2024).

30. S. B. Cohen, B. H. Gern, J. L. Delahaye, K. N. Adams, C. R. Plumlee, J. K. Winkler, D. R. Sherman, M. Y. Gerner, K. B. Urdahl, Alveolar macrophages provide an early *Mycobacterium tuberculosis* niche and initiate dissemination. *Cell Host Microbe* **24**, 439–446.e4 (2018).

31. A. O. Moguche, M. Musvosvi, A. Penn-Nicholson, C. R. Plumlee, H. Mearns, H. Geldenhuys, E. Smit, D. Abrahams, V. Rozot, O. Dintwe, S. T. Hoff, I. Kromann, M. Ruhwald, P. Bang, R. P. Larson, S. Shafiani, S. Ma, D. R. Sherman, A. Sette, C. S. Lindestam Arlehamn, D. M. McKinney, H. Maecker, W. A. Hanekom, M. Hatherill, P. Andersen, T. J. Scriba, K. B. Urdahl, Antigen availability shapes T cell differentiation and function during tuberculosis. *Cell Host Microbe* **21**, 695–706.e5 (2017).

32. D. Masopust, S. J. Ha, V. Vezys, R. Ahmed, Stimulation history dictates memory CD8 T cell phenotype: Implications for prime-boost vaccination. *J. Immunol.* **177**, 831–839 (2006).

33. M. D. Martin, V. P. Badovinac, Defining memory CD8 T cell. *Front. Immunol.* **9**, 2692 (2018).

34. D. Rai, M. D. Martin, V. P. Badovinac, The longevity of memory CD8 T cell responses after repetitive antigen stimulations. *J. Immunol.* **192**, 5652–5659 (2014).

35. N. Van Braeckel-Budimir, S. M. Varga, V. P. Badovinac, J. T. Harty, Repeated antigen exposure extends the durability of influenza-specific lung-resident memory CD8$^+$ T cells and heterosubtypic immunity. *Cell Rep.* **24**, 3374–3382.e3 (2018).

36. N. S. Joshi, W. Cui, A. Chandele, H. K. Lee, D. R. Urso, J. Hagman, L. Gapin, S. M. Kaech, Inflammation directs memory precursor and short-lived effector CD8$^+$ T cell fates via the graded expression of T-bet transcription factor. *Immunity* **27**, 281–295 (2007).

37. T. M. Onami, L. E. Harrington, M. A. Williams, M. Galvan, C. P. Larsen, T. C. Pearson, N. Manjunath, L. G. Baum, B. D. Pearce, R. Ahmed, Dynamic regulation of T cell immunity by CD43. *J. Immunol.* **168**, 6022–6031 (2002).

38. D. Amsen, R. A. Backer, C. Helbig, Decisions on the road to memory. *Adv. Exp. Med. Biol.* **785**, 107–120 (2013).

39. A. Kallies, K. L. Good-Jacobson, Transcription factor T-bet orchestrates lineage development and function in the immune system. *Trends Immunol.* **38**, 287–297 (2017).

40. T. Hussell, T. J. Bell, Alveolar macrophages: Plasticity in a tissue-specific context. *Nat. Rev. Immunol.* **14**, 81–93 (2014).

41. J. D. Yang, D. Mott, R. Sutiwisesak, Y.-J. Lu, F. Raso, B. Stowell, G. H. Babunovic, J. Lee, S. M. Carpenter, S. S. Way, S. M. Fortune, S. M. Behar, *Mycobacterium tuberculosis*-specific CD4$^+$ and CD8$^+$ T cells differ in their capacity to recognize infected macrophages. *PLOS Pathog.* **14**, e1007060 (2018).

42. L. Huang, E. V. Nazarova, S. Tan, Y. Liu, D. G. Russell, Growth of *Mycobacterium tuberculosis* in vivo segregates with host macrophage metabolism and ontogeny. *J. Exp. Med.* **215**, 1135–1152 (2018).

43. B. A. Norris, J. D. Ernst, Mononuclear cell dynamics in *M. tuberculosis* infection provide opportunities for therapeutic intervention. *PLOS Pathog.* **14**, e1007154 (2018).

44. R. Lai, T. Williams, T. Rakib, J. Lee, S. M. Behar, Heterogeneity in lung macrophage control of *Mycobacterium tuberculosis* is modulated by T cells. *Nat. Commun.* **15**, 5710 (2024).

45. Q. Qi, Y. Liu, Y. Cheng, J. Glanville, D. Zhang, J. Y. Lee, R. A. Olshen, C. M. Weyand, S. D. Boyd, J. J. Goronzy, Diversity and clonal selection in the human T-cell repertoire. *Proc. Natl. Acad. Sci. U.S.A.* **111**, 13139–13144 (2014).

46. P. A. Savage, J. J. Boniface, M. M. Davis, A kinetic basis for T cell receptor repertoire selection during an immune response. *Immunity* **10**, 485–492 (1999).

47. T. Cukalac, J. Chadderton, A. Handel, P. C. Doherty, S. J. Turner, P. G. Thomas, N. L. La Gruta, Reproducible selection of high avidity $CD8^+$ T-cell clones following secondary acute virus infection. *Proc. Natl. Acad. Sci. U.S.A.* **111**, 1485–1490 (2014).

48. M. Musvosvi, H. Huang, C. Wang, Q. Xia, V. Rozot, A. Krishnan, P. Acs, A. Cheruku, G. Obermoser, A. Leslie, S. M. Behar, W. A. Hanekom, N. Bilek, M. Fisher, S. H. E. Kaufmann, G. Walzl, M. Hatherill, M. M. Davis, T. J. Scriba, T cell receptor repertoires associated with control and disease progression following *Mycobacterium tuberculosis* infection. *Nat. Med.* **29**, 258–269 (2023).

49. K. L. Griffiths, M. Ahmed, S. Das, R. Gopal, W. Horne, T. D. Connell, K. D. Moynihan, J. K. Kolls, D. J. Irvine, M. N. Artyomov, J. Rangel-Moreno, S. A. Khader, Targeting dendritic cells to accelerate T-cell activation overcomes a bottleneck in tuberculosis vaccine efficacy. *Nat. Commun.* **7**, 13894 (2016).

50. E. Mata, R. Tarancon, C. Guerrero, E. Moreo, F. Moreau, S. Uranga, A. B. Gomez, D. Marinova, M. Domenech, F. Gonzalez-Camacho, M. Monzon, J. Badiola, J. Dominguez-Andres, J. Yuste, A. Anel, A. Peixoto, C. Martin, N. Aguilo, Pulmonary BCG induces lung-resident macrophage activation and confers long-term protection against tuberculosis. *Sci. Immunol.* **6**, eabc2934 (2021).

51. M. Jeyanathan, S. Afkhami, A. Khera, T. Mandur, D. Damjanovic, Y. Yao, R. Lai, S. Haddadi, A. Dvorkin-Gheva, M. Jordana, S. L. Kunkel, Z. Xing, CXCR3 signaling is required for

restricted homing of parenteral tuberculosis vaccine-induced T cells to both the lung parenchyma and airway. *J. Immunol.* **199**, 2555–2569 (2017).

52. Y. Yao, M. Jeyanathan, S. Haddadi, N. G. Barra, M. Vaseghi-Shanjani, D. Damjanovic, R. Lai, S. Afkhami, Y. Chen, A. Dvorkin-Gheva, C. S. Robbins, J. D. Schertzer, Z. Xing, Induction of autonomous memory alveolar macrophages requires T cell help and is critical to trained immunity. *Cell* **175**, 1634–1650.e17 (2018).

53. K. R. Renkema, M. A. Huggins, H. Borges da Silva, T. P. Knutson, C. M. Henzler, S. E. Hamilton, KLRG1$^{+}$ memory CD8 T cells combine properties of short-lived effectors and long-lived memory. *J. Immunol.* **205**, 1059–1069 (2020).

54. J. A. Olson, C. McDonald-Hyman, S. C. Jameson, S. E. Hamilton, Effector-like CD8$^{+}$ T cells in the memory population mediate potent protective immunity. *Immunity* **38**, 1250–1260 (2013).

55. K. H. Ely, A. D. Roberts, D. L. Woodland, Cutting edge: Effector memory CD8$^{+}$ T cells in the lung airways retain the potential to mediate recall responses. *J. Immunol.* **171**, 3338–3342 (2003).

56. M. Jeyanathan, A. Heriazon, Z. Xing, Airway luminal T cells: A newcomer on the stage of TB vaccination strategies. *Trends Immunol.* **31**, 247–252 (2010).

57. C. N. Horvath, C. R. Shaler, M. Jeyanathan, A. Zganiacz, Z. Xing, Mechanisms of delayed anti-tuberculosis protection in the lung of parenteral BCG-vaccinated hosts: A critical role of airway luminal T cells. *Mucosal Immunol.* **5**, 420–431 (2012).

58. R. S. Wallis, A. O'Garra, A. Sher, A. Wack, Host-directed immunotherapy of viral and bacterial infections: Past, present and future. *Nat. Rev. Immunol.* **23**, 121–133 (2023).

59. S. H. E. Kaufmann, A. Dorhoi, R. S. Hotchkiss, R. Bartenschlager, Host-directed therapies for bacterial and viral infections. *Nat. Rev. Drug Discov.* **17**, 35–56 (2018).

60. M. D. Muzumdar, B. Tasic, K. Miyamichi, L. Li, L. Luo, A global double-fluorescent Cre reporter mouse. *Genesis* **45**, 593–605 (2007).

61. S. Pai, J. Qin, L. Cavanagh, A. Mitchell, F. El-Assaad, R. Jain, V. Combes, N. H. Hunt, G. E. Grau, W. Weninger, Real-time imaging reveals the dynamics of leukocyte behaviour during experimental cerebral malaria pathogenesis. *PLOS Pathog.* **10**, e1004236 (2014).

62. H. D. Sathkumara, V. Muruganandah, M. M. Cooper, M. A. Field, M. A. Alim, R. Brosch, N. Ketheesan, B. Govan, C. M. Rush, L. Henning, A. Kupz, Mucosal delivery of ESX-1-expressing BCG strains provides superior immunity against tuberculosis in murine type 2 diabetes. *Proc. Natl. Acad. Sci. U.S.A.* **117**, 20848–20859 (2020).
